# Accelerating drug target inhibitor discovery with a deep generative foundation model

**DOI:** 10.1126/sciadv.adg7865

**Published:** 2023-06-21

**Authors:** Vijil Chenthamarakshan, Samuel C. Hoffman, C. David Owen, Petra Lukacik, Claire Strain-Damerell, Daren Fearon, Tika R. Malla, Anthony Tumber, Christopher J. Schofield, Helen M.E. Duyvesteyn, Wanwisa Dejnirattisai, Loic Carrique, Thomas S. Walter, Gavin R. Screaton, Tetiana Matviiuk, Aleksandra Mojsilovic, Jason Crain, Martin A. Walsh, David I. Stuart, Payel Das

**Affiliations:** ^1^ IBM Research, Thomas J. Watson Research Center, Yorktown Heights, New York, NY, USA.; ^2^ Diamond Light Source Ltd., Harwell Science and Innovation Campus, OX11 0DE Didcot, UK.; ^3^ Research Complex at Harwell, Harwell Science and Innovation Campus, OX11 0FA Didcot, UK.; ^4^ Chemistry Research Laboratory, Department of Chemistry and the Ineos Oxford Institute for Antimicrobial Research, University of Oxford, 12 Mansfield Road, OX1 3TA Oxford, UK.; ^5^ Division of Structural Biology, University of Oxford, The Wellcome Centre for Human Genetics, Headington, Oxford, UK.; ^6^ Wellcome Centre for Human Genetics, Nuffield Department of Medicine, University of Oxford, Oxford OX3 7BN, UK.; ^7^ Enamine Ltd., Chervonotkatska St, 67, Kyiv 02094, Ukraine.; ^8^ IBM Research Europe, Hartree Centre, Daresbury WA4 4AD, UK.; ^9^ Department of Biochemistry, University of Oxford, Oxford OX1 3QU, UK.

## Abstract

Inhibitor discovery for emerging drug-target proteins is challenging, especially when target structure or active molecules are unknown. Here, we experimentally validate the broad utility of a deep generative framework trained at-scale on protein sequences, small molecules, and their mutual interactions—unbiased toward any specific target. We performed a protein sequence-conditioned sampling on the generative foundation model to design small-molecule inhibitors for two dissimilar targets: the spike protein receptor-binding domain (RBD) and the main protease from SARS-CoV-2. Despite using only the target sequence information during the model inference, micromolar-level inhibition was observed in vitro for two candidates out of four synthesized for each target. The most potent spike RBD inhibitor exhibited activity against several variants in live virus neutralization assays. These results establish that a single, broadly deployable generative foundation model for accelerated inhibitor discovery is effective and efficient, even in the absence of target structure or binder information.

## Teaser

Inhibitors designed by a sequence-guided generative foundation model show broad utility and efficiency for multiple drug targets.

## INTRODUCTION

De novo molecular design, the proposing of previously unidentified compounds with desired properties, is a challenging problem with applications in drug discovery and materials engineering. For instance, a key objective in the drug discovery workflow is to identify candidate molecules that can interact with and inhibit a known drug-target protein and/or associated pathways with measurable activity. Searching for those inhibitor compounds that serve as the chemical starting points for further design of drug candidates typically involves high-throughput screening of libraries containing standard chemical compounds or smaller chemical fragments. Success rates for this method are between 0.5% and 1% (*1*), depending on the size of the library screened (typically on the order of 10^4^ entries) and target characteristics. This low success rate is in part due to the immense search space, now estimated to span between 10^33^ and 10^80^ feasible molecules (*2*), from which only a minute fraction typically has the traits sought. Exhaustive enumeration of this vast chemical space is infeasible, and prioritization of compounds to be screened is therefore challenging to perform with confidence.

In addition to the need for thousands of screening experiments, the initial selection of the library frequently requires detailed structural information on the target protein of interest bound to already reported ligands, which is often not readily available. Further, discovery is often performed using hand-crafted rules and heuristics to link existing fragments and/or to avoid impractical synthetic pathways. Many inhibitor discovery approaches tend to focus on compounds that have similar molecular structures to known inhibitors, whereas more promising compounds could be found in other, previously less explored, molecular structures. Finally, inhibitor discovery can be expensive, due to the cost of infrastructure, compounds, and reagents. Consequently, the cost of developing a single drug is high, reaching up to $2.8 billion, while the duration from concept to market typically exceeds a decade (*3*). Therefore, a more efficient approach is urgently needed, to enable distillation of previously unidentified and promising molecules from the vast chemical space, which includes molecules not yet synthesized. This approach will enable experimental validation of a small selection of candidates, resulting in a higher inhibitor discovery rate, at a reduced time and cost.

Deep learning–based generative models have the potential to enable discovery of previously unidentified molecules with desired functionality in a “rule-free” manner, as they aim to first learn a dense, continuous representation (hereafter referred to as a latent vector) of known chemicals and then modify the latent vectors to decode into unseen molecules. Such models thus offer access to previously unexplored chemical space unrestricted by conscious human bias. However, for the task of target-specific drug-like inhibitor design, an “inverse molecular design” (*4*) approach must be used, where the navigation through the learned chemical representation is guided by molecular property attributes, such as target inhibition activity and drug-likeness. In the case of designing inhibitors against a previously unidentified target, a sufficient amount of exemplar molecules is required, which is likely unavailable and requires costly and time-consuming screening experiments to obtain. As most existing deep generative frameworks [see Sousa *et al.* (*5*) for a review of generative deep learning for targeted molecule design] still rely on learning from target-specific libraries of binder compounds, they limit exploration beyond a fixed library of known and monolithic molecules while preventing generalization of the machine learning framework toward previously unidentified targets. As a result, while some studies (*6*–*8*) that use deep generative models for target-specific inhibitor design have been experimentally validated, to our knowledge, demonstrations of those models to tackle validated inhibitor discovery across dissimilar protein targets, without having access to detailed target-specific prior binding data (e.g., target binder molecules), have not been reported.

Our work demonstrates the real-world applicability of a single, unified inhibitor design framework, based on a deep generative foundation model, across different target proteins simultaneously. The generative framework only requires more readily available target sequence information to guide the design. Further, the work considers (i) off-target binding of the designed hits to account for potential downstream adverse effects, (ii) identifying hits even in the case of unknown binders, and (iii) prioritizing compounds that are readily synthesizable. We use CogMol (*9*), a deep generative model, to propose previously unidentified and chemically viable inhibitor designs for two important and distinct severe acute respiratory syndrome coronavirus 2 (SARS-CoV-2) targets—the receptor-binding domain (RBD) of the spike (S) protein and the main protease (M^pro^) protein. The deep generative framework, built upon large-scale data of chemical molecules, protein sequences, and protein-ligand binding data, serves as a generative foundation model for the target-aware inhibitor molecule design without any further fine-tuning on the target-specific data and can extrapolate to target sequences not present in the original training data. This broad generality of the CogMol framework therefore places it within the emerging class of “foundation models” (*10*, *11*), which are pretrained on a broad set of unlabeled data and can be used for different downstream tasks with minimal fine-tuning. A set of previously unidentified molecules targeting SARS-CoV-2 proteins, which were designed by CogMol, was shared under the Creative Commons license in April 2020 in the IBM COVID-19 Molecule Explorer platform (*12*). Here, we provide the first experimental validation of the broad utility and readiness of the CogMol deep generative framework, by synthesizing and testing the inhibitory activity of a number of prioritized designs against SARS-CoV-2 RBD of the S protein and M^pro^ protein. We further demonstrate the applicability of the binding affinity predictor model used in the CogMol framework by subjecting it to virtual screening of a library of lead-like chemicals and successfully identifying three compounds that were confirmed to be bound at the active site of the M^pro^ by crystallographic analysis, one of which showed micromolar inhibition.

To our knowledge, the present study provides the first validated demonstration of a single generative machine intelligence framework that can propose previously unidentified and promising inhibitors for different protein drug targets with a high success rate while only using protein sequence information during design. The demonstrated broad-spectrum antiviral activity of the designed spike inhibitor against the SARS-CoV-2 variants of concern (VOCs) further establishes the potential of such a deep generative framework to accelerate and automate the hit discovery cycle, a process known to suffer from low yield and high attrition rates, as well as advance our scientific understanding about less-explored drug targets.

## RESULTS

### Attribute-conditioned molecule generation with a deep generative model

**Fig. 1.**
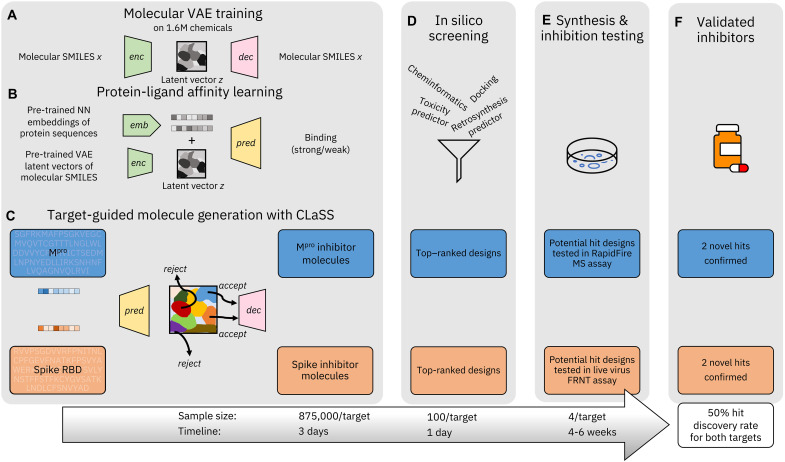
Overview of our inhibitor discovery workflow driven by CogMol, a sequence-guided deep generative foundation model. (**A** and **B**) Molecular Variational AutoEncoder (VAE) training on large-scale chemical SMILES (**x**) data and mapping of existing protein-ligand affinity relations on the VAE latent space (**z**) by training a binding predictor, respectively. For the latter, we leverage pretrained neural network (NN) embeddings of a large volume of protein sequences. (**C**) Schematic representation of Controllable Latent (attribute) Space Sampling (CLaSS), which samples from the model of VAE latent vectors by using the guidance from a set of molecular property predictors (e.g., protein binding) such that, for a given target protein sequence, sampled **z** vectors corresponding to strong target binding affinity are accepted, while vectors corresponding to weak target binding affinity are rejected. The accepted **z** vectors are then decoded into molecular SMILES. (**D**) Candidates are then ranked and filtered according to chemical properties, docking score to target structure, and predicted retrosynthetic feasibility and toxicity. (**E**) A small set of prioritized molecules are synthesized, followed by wet laboratory testing in specific in vitro assays to confirm target inhibition. (**F**) In the present case, for each target, of the four molecules tested, two showed promising levels of inhibition. The hit discovery rate reported is therefore the fraction of the AI-designed compounds that were synthesized and experimentally tested, which showed inhibition in target-specific assays. We also report approximate sample sizes and timeline for each stage of our discovery workflow. Note the timeline does not include the training and testing of the generative and predictive machine learning models.

The overall inhibitor discovery pipeline is described in Fig. 1 and consists of three main steps: (A to C) candidate design in a target-conditioned manner using the deep generative framework, (D) in silico screening for candidate prioritization, and (E) wet laboratory validation of prioritized molecules. For de novo molecule design, we used the deep generative framework CogMol as a foundation, which enables the design of inhibitor molecules for different targets, without requiring training or fine-tuning the model on target-specific data. Hereafter, we refer to machine-designed previously unidentified compounds as de novo compounds throughout the rest of the paper.

CogMol works as follows: First, it uses a variational autoencoder (VAE) (*13*), a popular class of deep learning–based generative models, as the generative foundation (Fig. 1A). A VAE is composed of a pair of neural nets—the encoder-decoder pair. The encoder neural network maps the simplified molecular-input line-entry system (SMILES) (*14*) string of a molecule into a low-dimensional representation. We will denote the encoder as *q*_ϕ_(**z**∣**x**), where **z** is a latent encoding of input SMILES **x** and ϕ represents the encoder parameters. The decoder *p*_θ_(**x**∣**z**), which is also a neural network, then converts the latent vector **z** back into the reconstructed SMILES **x**. The encoder in a VAE is probabilistic in nature as it outputs latent encodings that are consistent with a Gaussian distribution. The decoder is therefore stochastic—it samples from the latent distribution to produce an output **x**. The encoder-decoder pair is trained end-to-end to optimize two objectives simultaneously. The first objective includes minimizing a loss term to ensure accurate reconstruction of an input SMILES from the corresponding latent embedding. The second objective consists of a regularization term to constrain the latent encodings to a standard normal distribution. The resulting latent space is continuous, enabling smooth interpolation as well as random sampling of diverse molecules from the latent space. To learn meaningful latent molecular representations that have general knowledge about diverse chemicals, in CogMol the VAE is trained on more than 1.6 × 10^6^ small molecules from public databases (see Materials and Methods for details).

Once the chemical latent representation is learned, CogMol performs attribute-conditioned sampling on that representation to generate unseen molecular entities with properties biased toward the design specifications. Specifically, the goal is to design drug-like molecules with a high binding affinity to the target protein of interest. Two **z**-based property predictors are used: a quantitative estimate of drug-likeness (QED) predictor and a target-molecule binding (strong/weak) predictor. Both predictors used the **z** encodings of molecules as input. For the binding predictor, the protein sequence embeddings from a preexisting deep neural net (*15*) was concatenated with the molecular latent encodings and trained on the general protein-molecule binding affinity data available in the BindingDB database (Fig. 1B). Performance of the attribute predictors is reported in Materials and Methods.

Given a target protein sequence of interest, those two predictors are used together to sample molecules with desired properties from the latent space, by using the CLaSS sampling method proposed by Das *et al.* (*16*). CLaSS relies on a rejection sampling schema to accept/reject molecules while sampling from a density model of the **z** embeddings. Acceptance/rejection criteria are determined by the output probabilities of property predictors. See Materials and Methods and algorithm S1 for further details on CLaSS.

Note, the CogMol generative framework relies on a chemical VAE, a protein sequence encoder, and a set of molecular property predictors, all of which are pretrained on large amount of broad data—i.e., chemical SMILES, protein sequences, and available protein-ligand binding affinities. The generative framework thus has important information already encoded about protein sequence homologies, chemical similarities, and protein-drug binding relations. This allows the framework to serve as a foundation, as it is instantly adaptable to different targets, without any further model retraining or fine-tuning on target-specific data. The approach further saves time and cost associated with generating target-specific binder libraries or resolving the target structure, which are typically considered as privileged information, i.e., not broadly available. The model can also extrapolate to a target that does not share high similarity with the training data. This is indeed the case for the SARS-CoV-2 targets considered (see table S1) where the lowest Expect value, a measure of sequence homology (lower values indicate high homology), with respect to the BindingDB protein sequences is 0.51 (query coverage = 40%) and 1.9 (query coverage = 26%) for M^pro^ and spike RBD, respectively. This analysis implies that both targets are not substantially similar to the protein sequences in the BindingDB database that was used for training the binding predictor, spike RBD being more distinct than M^pro^; nor do they share any substantial sequence, structure, or functional similarity to each other.

### Candidate prioritization from the machine-designed ligand library

The next stage includes in silico screening of generated candidates (Fig. 1D) to prioritize them for synthesis and wet laboratory evaluation. For practical considerations, we sought to keep the number of prioritized machine-designed de novo compounds to be synthesized and tested very small—around 10 for each target, as opposed to screening thousands of existing chemicals in a more traditional setup, as synthesis of previously unknown chemicals is costly and time-consuming, particularly during a global pandemic. Careful analysis, including machine learning–based retrosynthesis predictions, was conducted to define this set. We used a combination of physicochemical properties (estimated using cheminformatics), target-molecule binding free energy predicted by docking simulations, and retrosynthesis and toxicity predictions by using machine learning. For retrosynthesis prediction, we used the IBM RXN platform (*17*) that is based on a transformer neural network trained on chemical reaction data. For toxicity prediction, an in-house neural network–based model trained on publicly available in vitro and clinical toxicity data was used. See Materials and Methods for details of candidate filtering and prioritization criteria. At the end of the in silico screening, the number of candidates per target was around 100, which was further narrowed down to around 10 per target by using the discretion of Enamine Ltd., the chemical manufacturer. Feasibility of the predicted reaction schema, as evaluated by organic synthetic chemist experts, as well as commercial availability and cost of the predicted reactants, was used to finalize the candidate synthesis list. The final four candidates for each target were chosen based on the synthesis cost and delivery time, as provided by Enamine.

### Synthesis of de novo compounds

**Fig. 2.**
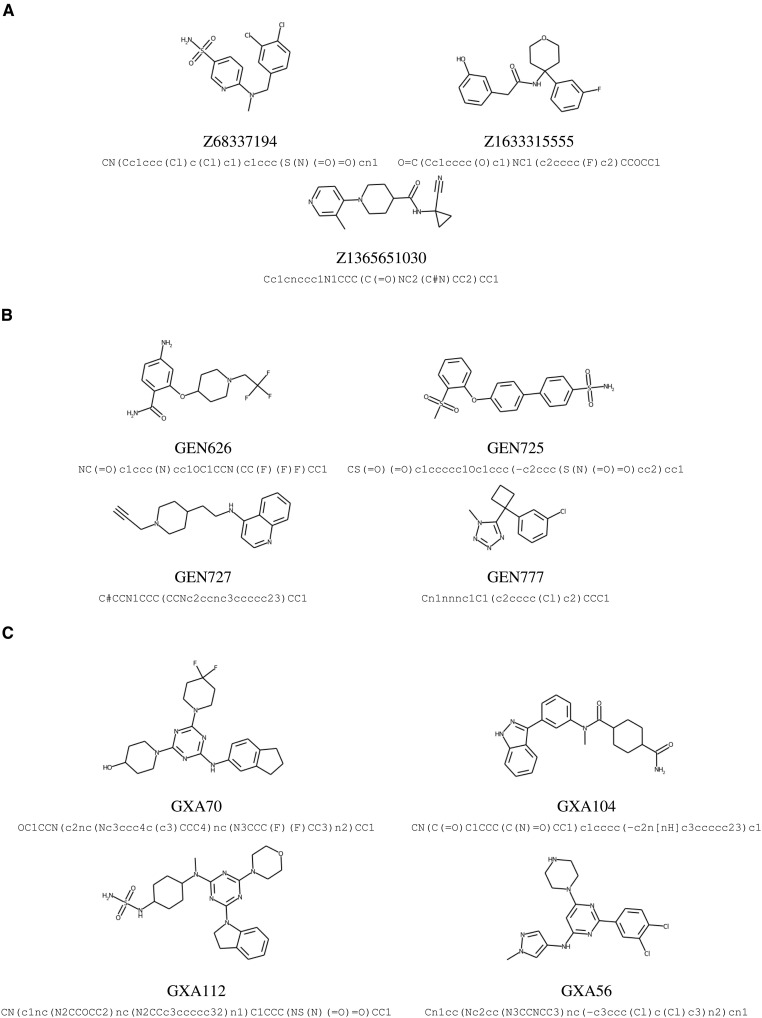
De novo designed and commercially sourced molecules. (**A**) Molecules with the prefix “Z” are molecules from the Enamine Advanced Collection catalog targeting M^pro^. Molecules with the prefix “GEN” are generated candidates targeting the spike RBD (**B**), while those with the prefix “GXA” are generated candidates targeting M^pro^ (**C**).

Figure 2 lists the eight de novo compounds designed by the generative machine learning framework that were synthesized (see tables S2 and S3 for the predicted molecular properties). Details of the experimental synthesis protocols are provided in Materials and Methods and figs. S1 to S3. We also provide a comparison between the predicted and the actual retrosynthetic pathways for those eight machine-designed compounds in table S4. Five were synthesized using the top predicted pathway of IBM RXN. For two compounds, GEN626 and GEN777, predictions were found to be unsuccessful, so alternative pathways as designed by Enamine were used (see Materials and Methods for details). For GXA104, reactants included in the RXN prediction were not available, so an alternative route was used. Overall, these results show the usefulness of machine learning–based retrosynthesis predictions for reliably identifying plausible candidates and recommending viable synthesis routes.

### De novo RBD-targeting compounds exhibit spike-based pseudovirus and live virus inhibition

**Fig. 3.**
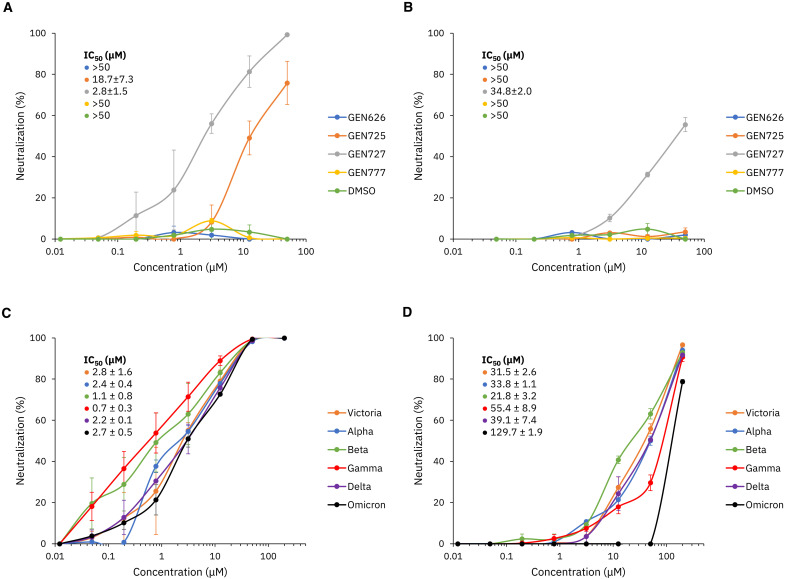
SARS-CoV-2 spike neutralization assays. Neutralization assay against SARS-CoV-2 pseudotyped lentivirus (**A**) and Victoria live virus (**B**) for four CogMol-generated compounds with DMSO as a control. (**C**) The most effective compound, GEN727, was selected for a pseudoviral neutralization assay against Victoria, Alpha, Beta, Gamma, Delta, and Omicron variants of concern (VOCs), as well as (**D**) the live virus neutralization assay. Error bars show the standard error of each measurement over two trials.

For the CogMol-designed compounds targeting the spike RBD, we measured their neutralization ability using a spike-containing pseudotyped lentivirus and a live viral isolate. These results are summarized in Fig. 3. Out of the four candidates, GEN725 and GEN727 showed IC_50_ (half-maximal inhibitory concentration) values less than 50 μM (18.7 and 2.8 μM, respectively), indicating discovery of previously unidentified hits with reasonable inhibition of the pseudovirus at a 50% success rate (Fig. 3A). GEN727 exhibited live virus neutralization ability as well (Fig. 3B).

We further checked if GEN727 is effective across different SARS-CoV-2 variants. We compared the neutralization of viral VOCs—Alpha, Beta, Delta, and Omicron—with neutralization of Victoria (SARS-CoV-2/human/AUS/VIC01/2020), a Wuhan-related strain isolated early in the pandemic from Australia, in both pseudovirus and live virus. Figure 3C shows that GEN727 neutralizes spike-containing pseudovirus across all VOCs with an IC_50_ value between 0.7 and 2.8 μM. Live virus data also show inhibition with an IC_50_ of less than 50 μM for Victoria, Alpha, Beta, and Delta (Fig. 3D).

The virus neutralization results do not demonstrate direct interactions of GEN727 with the spike. To probe this, we performed thermofluor measurements to determine if GEN727 affected the stability of the spike. The presence of the compound appeared to reduce the speed of the transition of the spike to a less stable form; after overnight incubation at pH 7.5, very little of the spike population remained in the more stable form with the higher *T*_m_ of 65°C (see fig. S9).

### De novo spike inhibitors target the conserved lipid-binding pocket in RBD

**Fig. 4.**
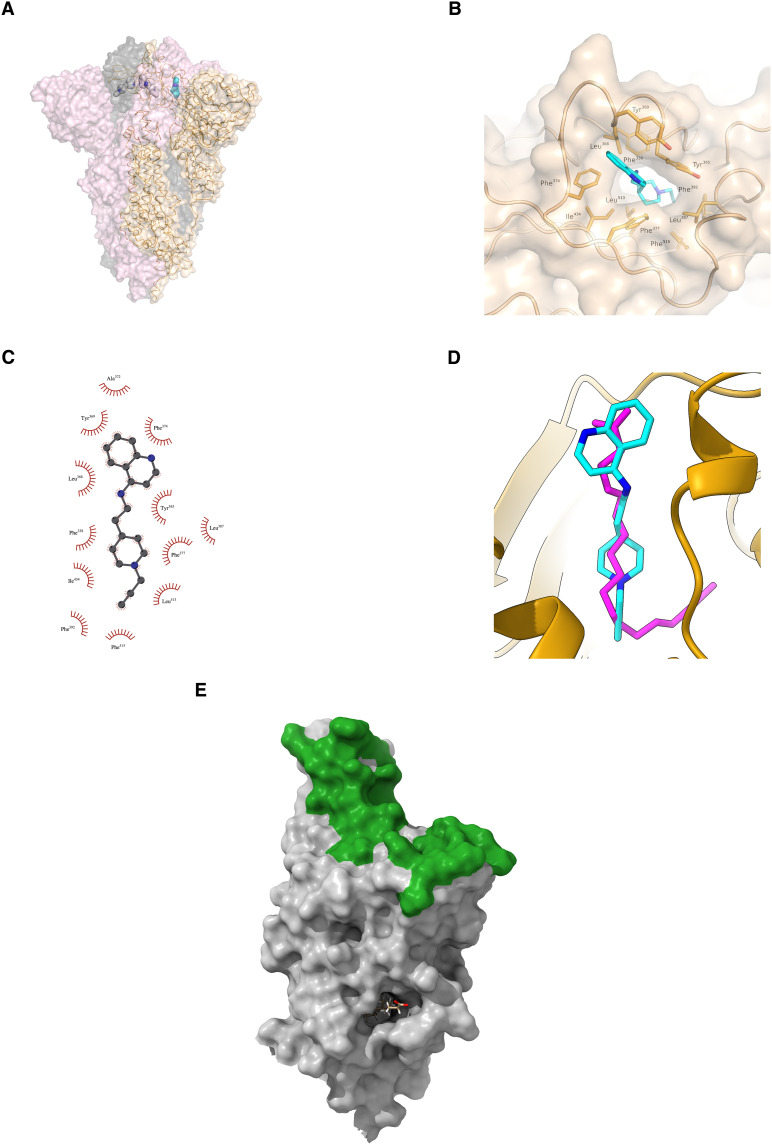
Docked structure of SARS-CoV-2 spike protein RBD in complex with GEN727. (**A**) Ribbon representation with transparent surface of the spike trimer. Wheat, gray, and light pink color is used to delineate each protomer. GEN727 (shown in stick representation) docked to a spike monomer structure is superimposed for reference. (**B**) Surface representation depicting the overall docking pose of GEN727 at the lipid binding site of the spike RBD. (**C**) Schematic of GEN727 interacting with the RBD. (**D**) Docked GEN727 (cyan) in reference to stearic acid lipid (magenta) bound to the spike RBD. (**E**) Stearic acid binding pocket. Stearic acid (shown as sticks, almost completely buried) is distant from the sites of binding of most neutralizing antibodies, which attach much higher up the molecule, overlapping the site of attachment of ACE2 (the green surface) and thereby blocking attachment to the host cell.

It should be noted that the detailed binding pose data have been impossible to obtain for the RBD with either of the de novo inhibitors. Therefore, we used in silico simulations to provide insight into the plausible binding modes. We first perform docking simulations on the generated molecules in the presence of spike RBD [Protein Data Bank (PDB) ID: 7Z3Z; see Materials and Methods for details]. Figure 4 reveals that GEN727 contacts with several tyrosines and hydrophobic residues, such as Tyr^365^, Tyr^369^, and Phe^374^, from RBD. Docking simulations revealed an interaction pattern similar to that of GEN727 for GEN725 as well (see fig. S10). Those tyrosines and phenylalanines constitute the lipid-binding pocket of the spike RBD. It has been found that the pocket is conserved across seven coronaviruses that infect humans and, therefore, may offer a target for broadly active antiviral agents (*18*). This pocket is distant and distinct from the sites of binding of the vast majority of neutralizing antibodies, which cluster at the site of ACE2 binding (see Fig. 4E). Binding of the lipid has been reported to stabilize the closed form of the spike, thereby interfering with receptor interactions, virion attachment, and subsequent host cell entry (*18*–*20*). Further, the lipid binding is considered “a conserved hallmark in pathogenic β-coronavirus spike proteins from SARS-CoV to Omicron” and experiments show that lipid treatment of cells that are already SARS-CoV-2–infected substantially reduces the production of virions or induces deformity in the produced virions (*21*). Recently, nuclear magnetic resonance experiments (*22*) have confirmed binding of a drug molecule Lifitegrast to the lipid binding pocket, which is used to treat symptoms of dry eye and has shown dose-dependent antiviral potency against SARS-CoV-2 in vitro (*23*). Earlier in vitro experimental study showed no substantial direct interaction between the lipid molecule and human ACE2 (*24*), consistent with the lower docking score (−8.1 kcal/mol for RBD versus −7.4 kcal/mol for human ACE2) between GEN727 and ACE2 found in our calculations. Also, docking reveals attachment of GEN727 to ACE2 at a site, which is far from the spike binding site (see fig. S11). GEN727 is also quite dissimilar to Lifitegrast, with a Tanimoto similarity of only 0.113.

**Fig. 5.**
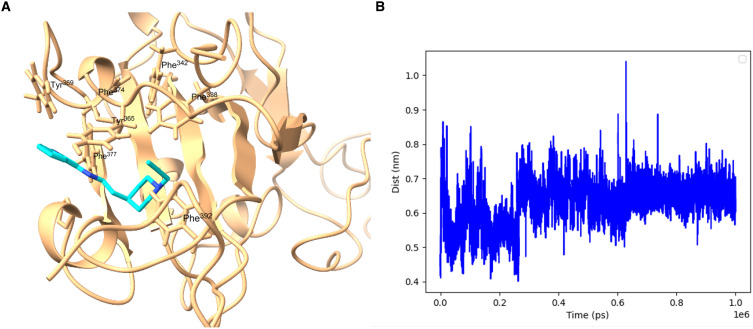
Model of GEN727 in the lipid binding pocket of SARS CoV-2 RBD. (**A**) Snapshot from MD simulation at the end of 1 μs. (**B**) Plot of protein-ligand distance [between the center of mass of GEN727 (shown in cyan/blue) and the center of mass of the lipid binding pocket, heavy atom only, in nm], as a function of simulation time (in ps). The lipid binding pocket is defined by five Phe residues, Phe^338^, Phe^342^, Phe^374^, Phe^377^, and Phe^392^.

The docking of GEN727 strikingly recapitulates the binding of the natural lipid (see Fig. 4D), suggesting that the lipid binding function maintains the conserved site targeted by GEN727. Further insight into GEN727 binding to the lipid binding site comes from molecular dynamics (MD) simulations (see Materials and Methods). Figure 5 shows stable interactions between GEN727 and conserved residues from the lipid binding pocket throughout 1 μs of simulation time. Consistently, the total GEN727-RBD interaction energy estimated from the MD simulation is −46.68 ± 0.64 kcal/mol.

The failure to experimentally resolve the binding pose of GEN727 likely stems from the fact that GEN727 binds to the lipid binding pocket. As a consequence of the biological function, the pocket becomes occupied at an early stage in the formation of the particle. Once the particle is released, there is a rather rapid conversion to a more open form and the loss of lipid. This conversion is not reversible, and we have been unable to rebind the natural lipid or any other compound. The viral and pseudoviral assays circumvent this by allowing substitution of the lipid by the compound (and subsequent blocking of the virus replication cycle) at an earlier stage in the virus life cycle than we can capture in our structural studies. Nevertheless, the thermofluor results (fig. S9) showed an (albeit weak) indication that incubation of spike with GEN727 somewhat destabilized the spike, suggestive of a direct interaction underlying its broad-spectrum neutralization ability.

The viral and pseudoviral neutralization experiments and the simulation findings reported in this study, together with the recent literature, thus further open the door for a lipid-mimetic antiviral intervention strategy, as a small molecule, which is designed by artificial intelligence (AI) and putatively targets the conserved lipid binding pocket, shows broad-spectrum antiviral activity for the first time.

### M^pro^ inhibition of de novo and commercially sourced compounds

**Fig. 6.**
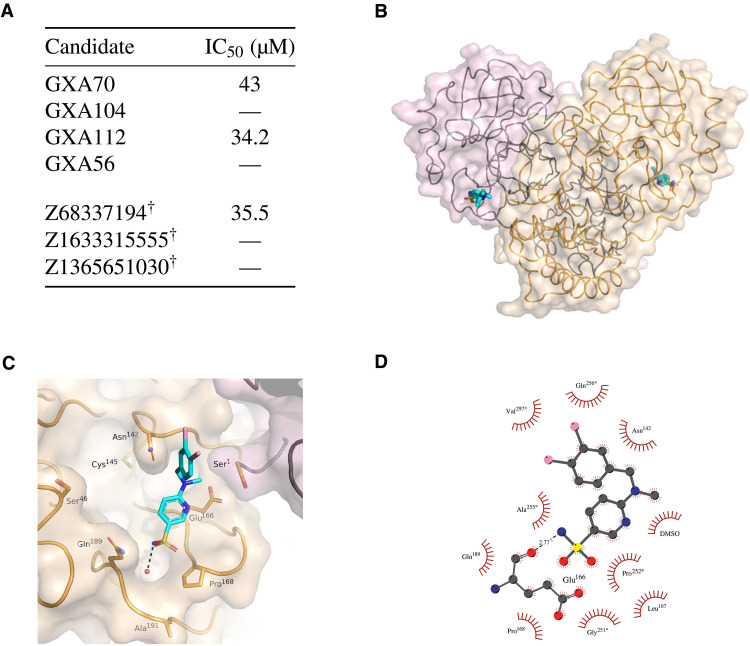
Inhibition of SARS-CoV-2 M^pro^ by machine-designed de novo and commercially sourced compounds. (**A**) Half-maximal inhibitory concentration (IC_50_) from RapidFire MS experiments for de novo and commercial M^pro^ inhibitor candidates. Symbol “—” indicates that no inhibition was detected. Candidates marked with ^†^ had successful crystal structures determined. (**B** to **D**) Crystal structure of the SARS-CoV-2 M^pro^ in complex with Z68337194. (B) Ribbon representation with transparent surface of the M^pro^ dimer colored in wheat and light pink to delineate each protomer. The active site of each protomer is shown with Z68337194 in stick representation. (C) Surface representation showing the overall binding mode of Z68337194 at the active site of M^pro^. (D) Schematic representation of the interactions of Z68337194 with M^pro^. Residues indicated with * are from a symmetry-related M^pro^ protomer.

To establish the generalizability and readiness of our framework, we next provide experimental validation results of the four prioritized de novo M^pro^ inhibitor candidates. Enzymatic inhibition by the CogMol-designed M^pro^-specific molecules was measured by solid-phase extraction purification linked to mass spectrometry (RapidFire MS) (*25*). The results are presented in Fig. 6A. Out of the four de novo compounds tested for this target, GXA70 and GXA112 both showed M^pro^ inhibition in the micromolar range, with IC_50_ values of 43 and 34.2 μM, respectively. These data are considered to be a good baseline for initial hit discovery, similar to those used in the prior studies (*7*, *26*–*29*). This, again, implies a 50% success rate of hit discovery for M^pro^. It is important to note that prior studies do leverage knowledge of existing active molecules, which is not the case in the present work, as the goal here is to simulate the scenario of targeting less explored proteins. Further, although target structure–based docking simulations are used for candidate prioritization, the inhibitor design by inferring from CogMol relies only on the target protein sequence and does not need detailed characterization of the ligand binding pocket within the target protein. This advantage is useful while designing inhibitors for previously unidentified targets and/or little-known binding pockets in a well-studied target.

We further tested the generalizability of the pIC_50_ predictor (trained directly on the molecular SMILES and protein sequences) by validating predictions on selected commercially available lead-like compounds from the Enamine Advanced Collection (*30*). For this purpose, we selected the top three Enamine compounds based on their predicted pIC_50_. One of these Enamine compounds showed inhibition (IC_50_ = 35.5 μM). Based on these results, we cocrystallized M^pro^ in the presence of this compound (ID Z68337194) and successfully obtained crystals (see table S5). The crystal structure determined revealed Z68337194 bound in the active site pocket. Structures of the other two commercially available compounds selected based on the pIC_50_ predictions were also found bound to the active site of M^pro^, although these compounds showed no detectable inhibition of M^pro^ using the RapidFire MS–based assay.

### Insights into the binding mode of the M^pro^ inhibitors

Detailed analysis of the structure obtained for the complex of M^pro^ with Z68337194 (see Fig. 6, B to D) reveals that the sulfonamide group sits in the P4 subsite (*31*) and the amine forms an electrostatic interaction with the backbone carbonyl of Glu^166^. This interaction mimics that made by the P4 site amide of nirmatrelvir (PF-07321332) (see fig. S12) (*32*). Z68337194 occupancy refines to approximately 50%. In the active site, shifts are observed in the positions of Pro^168^, Leu^167^, Glu^166^, and Met^165^ to accommodate ligand binding. The compound does not sit deeply in the active site and does not interact with the catalytic machinery, providing opportunities to elaborate upon the compound to take advantage of further subsites. In the captured crystal form, the active site sits at the interface between symmetry-related protein monomers, and as a result, a symmetry-related molecule provides additional interactions—primarily a stacking interaction between the ligand phenylamine ring and Pro^252^. Additionally, a hydrophobic pocket in the symmetry mate formed primarily by Gln^256^ and Val^297^ accommodates the chlorinated ring.

**Fig. 7.**
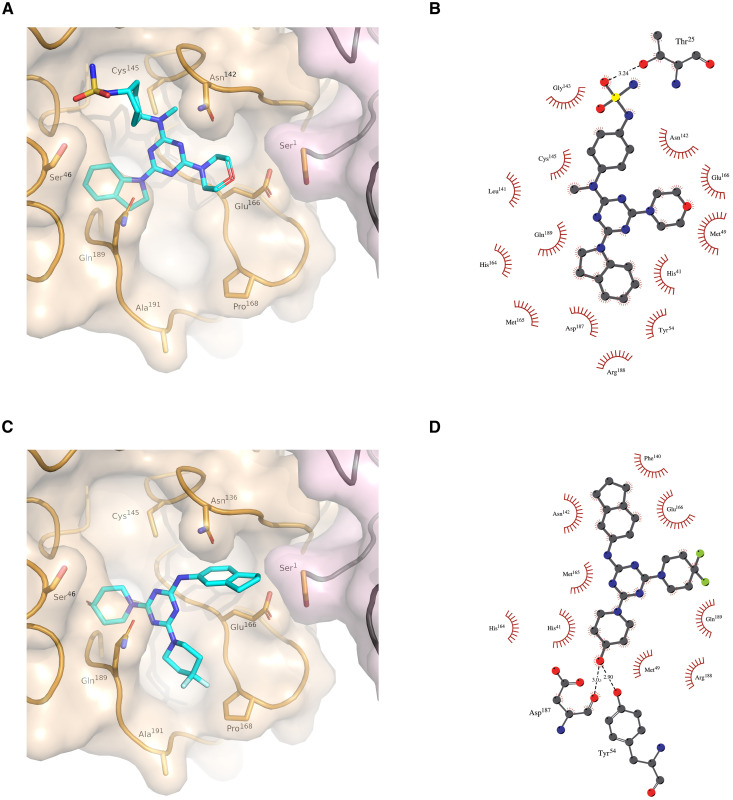
Docked structures of SARS-CoV-2 M^pro^ with GXA112 and GXA70. Surface representation depicting the overall ligand binding modes of (**A**) GXA112 and (**C**) GXA70 at the active site of M^pro^. Schematic representation of the ligand interactions with M^pro^ for (**B**) GXA112 and (**D**) GXA70.

As experimental determinations of the structure of M^pro^ in complex with the validated de novo inhibitors were not fruitful, we used docking simulations to provide insight into the plausible binding modes with the target structure [PDB ID: 6LU7 (*31*) for M^pro^]. As shown in Fig. 7, both GXA112 and GXA70 revealed mainly hydrophobic contacts to the residues from the P1 and P2 subsites, which are the hotspots of interactions (*31*). The hydrogen bonding pattern revealed by the two molecules is, however, starkly different: GXA112 forms hydrogen bonding mainly with P1′ site (T25), whereas GXA70 interacts with the P2 residues (D187 and Y54). The nonextensive and diverse interaction pattern of the de novo and commercially sourced M^pro^ inhibitors reported in this study is consistent with reported observations for noncovalent inhibitors (*33*).

### Novelty of the de novo designs and comparison with known SARS-CoV-2 inhibitors

**Fig. 8.**
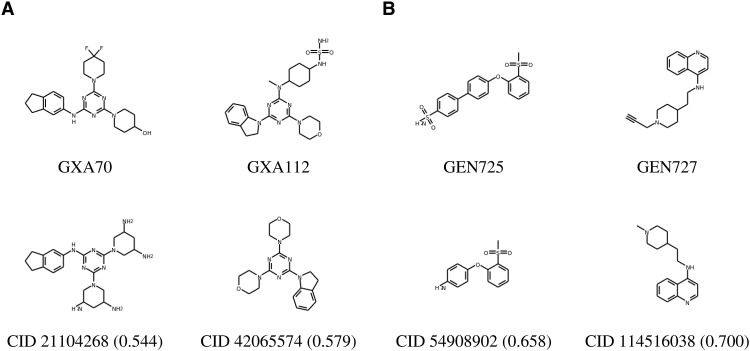
Molecular similarity with PubChem compounds. Top: Validated de novo compounds targeting (**A**) M^pro^ and (**B**) spike RBD. Bottom: Most similar molecules from PubChem. Values in parenthesis indicate Tanimoto similarity between the machine-designed and nearest PubChem molecules.

To characterize the novelty of the de novo bioactive hits, we identified the nearest compound from the PubChem database, in terms of their Tanimoto similarity (*34*) estimated using Morgan fingerprints (*35*). Figure 8 reveals that none of the de novo molecules shares ≥0.7 Tanimoto similarity with PubChem molecules. For additional analyses, see table S6.

### Drug-likeness analysis of validated hits

Finally, we have predicted the drug-like nature and the medicinal chemistry friendliness of the experimentally validated hits found in this study, which are either de novo designed or commercially available. For this purpose, the SwissADME (*36*) software was used. Table S7 provides a summary of those results (complete analysis reports can be found in figs. S13 to S18), suggesting that the inhibitor hits satisfy several typical properties of drug-like, orally bioavailable compounds in terms of the drug-likeness scores and bioavailability. Further, the compounds rarely contain any medicinal chemistry alerts and fulfill most criteria for lead-likeness. These analyses are consistent with low predicted toxicity and positive target selectivity (see Materials and Methods and tables S2 and S3). Such favorable bioavailability and drug-likeness suggest potential of these inhibitor hits as a starting point for further optimization toward compounds with more potency and better pharmacokinetic properties, by using various machine learning–based optimization techniques (*37*) and/or medicinal chemistry approaches.

## DISCUSSION

The discovery of drug-target protein inhibitors has been greatly advanced by the combined power of numerous in silico approaches. Nevertheless, even the most effective methods face broad challenges that are at the same time inherent to general inverse molecular design tasks and specific to biological target-ligand binding chemistry. The first of these pertains to the vastness of the chemical space being explored and its impact on the throughput and practical utility of the prevailing methods. For example, the use of docking or molecular simulation methods to screen on the order of 10^8^ to 10^9^ commercially available compounds would incur a prohibitively high computational cost, estimated to reach 10 central processing unit (CPU) years (*38*) per target (as opposed to screening of less than a thousand machine-designed de novo candidates via docking in the present study).

The second challenge is availability of critical information: While methods such as pharmacophore modeling and molecular docking and simulations have been used successfully in virtual screening or design of molecules (*26*–*28*, *38*–*40*), such approaches generally rely upon initial design constructs obtained from available crystal structure(s) of a target protein bound to a candidate compound or fragment hits. For example, Glaab *et al.* (*27*) have reported experimental validation of computationally screened M^pro^ inhibitors: out of 95 candidates tested in vitro, 2 showed IC_50_ values less than 50 μM. A variety of different computational approaches were used for screening: (i) searching for the nearest neighbors of a known M^pro^ inhibitor, (ii) M^pro^ structure-based screening using molecular docking followed by molecular simulations, and (iii) binding prediction using a machine learning model trained on existing M^pro^ binders and nonbinders. Such knowledge of structures bound to known inhibitors is not guaranteed to be available for all drug targets of interest and may take months to derive experimentally, and consequently, these approaches are not broadly applicable to the case where target structures or inhibitors are unknown. Recently, the field of structural biology has been revolutionized by deep learning–based methods [e.g., AlphaFold (*41*) and RoseTTAFold (*42*)] for predicting the three-dimensional structure of a protein from its sequence. While they predict structures with often astonishing accuracy, the structural models derived from neural networks are still relatively limited in aiding the understanding of natural protein function, in particular understanding the interactions with protein partners or small ligands. Therefore, the deduction of functional ligand and drug interaction still remains predominantly reliant on resource-intensive experimental (bio)chemical techniques, e.g., assays, structural determination, and synthesis.

In general, reliance on privileged information (the target protein structure and/or known hits) confines the discovery space to the neighborhood of known chemical entities (*27*). This dependency therefore presents a practical challenge to expand the accessible chemical exploration space and to devise more readily generalizable approaches to inhibitor design for multiple targets, the structure and binders of which may not be known.

Previous generative machine learning models that have been subject to experimental validation of de novo–designed molecules were primarily either trained or fine-tuned on a target-specific ligand library (*6*, *7*, *43*–*47*). This work establishes the basis for an alternative discovery paradigm, wherein a generative model is used to discover previously unidentified inhibitor hits for different protein targets in an automated fashion. To our knowledge, this is the first validated demonstration of a single generative model enabling successful and efficient discovery of drug-like inhibitor molecules for two very different target proteins, based only on the protein sequence that is used during model inference. The generation of previously unidentified, drug-like, target-specific inhibitor molecules is automated, as the approach performs attribute-controlled sampling on the learned abstract molecular representation space and does not rely on virtual screening of generated compounds that were designed using cumbersome rule-based fragmentation [e.g., as in Morris *et al.* (*26*)]. Moreover, to our knowledge, none of the earlier studies considers the challenging, but highly practical, scenario of designing and experimentally validating inhibitors for several distinct targets in parallel, without using the target binder information, which resembles the scenario of relatively unknown targets. Further, to our knowledge, evaluation of AI-generated retrosynthesis pathway predictions against wet laboratory compound production has not been reported at this scale for AI-designed, previously unidentified inhibitors. Learning from comparisons between actual and predicted synthesis pathways can help the AI model to be more accurate and provide better coverage.

The sequence information of previously unidentified drug targets typically emerges at a much faster (days versus months) pace than their detailed structural information, thanks to the latest advances in sequencing. The structural deduction of target-ligand interaction takes even longer. In contrast, as shown in Fig. 1, it took us less than a week to design and prioritize the set of candidate molecules to be synthesized and tested in wet laboratory for the two SARS-CoV-2 targets, as our approach does not reply on target structure or binder information. The information on SARS-CoV-2 sequences was made publicly available starting around January of 2020, and CogMol-designed candidates were open-sourced in the IBM COVID-19 Molecule Explorer platform in April 2020. While the prioritized de novo compounds were ordered in August 2020, the first round of wet laboratory validation was completed in October 2020. This rapid pace of previously unidentified drug-like inhibitor discovery across two distinct drug targets, when the world was experiencing a pandemic, shows the potential of a sequence-guided generative machine learning–based framework to help with better pandemic preparedness and other global emergencies.

The overall success rate of inhibitor discovery found here is 50% for both targets, which required synthesizing and screening only four compounds per target. In addition, one of the three commercially sourced compounds also showed M^pro^ inhibition. This result shows promise of the proposed approach, particularly when compared to a <10% hit discovery obtained typically using high-throughput screening (*1*, *27*). Additionally, the validated de novo inhibitors reported in this study appear to be distinct, based on molecular similarity analyses with existing chemicals and SARS-CoV-2 inhibitors, indicating substantial creativity by the generative framework, which is not possible when screening known compounds. The compounds also satisfy criteria of drug-likeliness and bioavailability. The efficiency of inhibitor discovery realized here and the demonstrated generalizability to distinctly dissimilar targets advocate for pretraining on a large volume of general data, e.g., chemical SMILES, protein sequences, and protein-ligand binding affinities. Conceptually, this is a key feature of so-called foundation models (*10*, *11*), which are trained on broad data at scale and can be easily adapted to many tasks. This perspective is also consistent with the recent work, establishing the informative nature of a deep language model trained on a large number of protein sequences, in terms of capturing fundamental properties (*16*, *48*). Thus, the validation of the framework reported here satisfies the generally accepted criteria of a foundation model, in the sense that it is trained on a broad set of unlabeled data, without a specific bias to a particular target, and is applicable without little or no fine-tuning to the general target-specific inhibitor discovery problem. The broad-spectrum efficacy across SARS-CoV-2 VOCs of the most potent spike hit observed is a further example of the foundational aspect of the model: The VOC sequences were never made available to the generative framework during training or inference. Moreover, to our knowledge, this is the first report of a previously unidentified spike-based noncovalent inhibitor that exhibits broad-spectrum antiviral activity. This contrasts with therapeutic monoclonal antibodies (mAbs), the only drugs currently in use that target the spike protein, where rather few are effective across VOCs (*49*). While the mutability of the spike is obvious because of the pressure to escape antibody neutralization, the widespread use of small-molecule drugs will also apply a strong pressure—as seen for instance in the rapid development of resistance to the first generation of anti–HIV-1 drugs. The choice of a binding site that is likely to be preserved to maintain a biological function, as seems to be the case with the RBD lipid pocket, is probably about the best we can do in the early stages of drug discovery to build in some resilience.

Together, the results presented here establish the efficiency, generality, scalability, and readiness of a generative machine intelligence foundation model for rapid inhibitor discovery against existing and emerging targets. Such a framework, particularly when combined with autonomous synthesis planning and robotic synthesis and testing (*8*), can further enhance preparedness for novel pandemics by enabling more efficient and precise antiviral design, which can chart a path toward better therapeutics. The generality and efficiency of the mechanisms used in CogMol for precisely controlling the attributes of generated molecules, by plugging in property predictors post hoc to a learned chemical representation, makes it suitable for broader applications in advancing molecular and material discoveries. For example, a similar framework has already enabled previously unidentified photoacid generator molecule design in a data-efficient manner for performant and sustainable semiconductor manufacturing, which has been validated by subject matter experts (SMEs) (*50*).

There remains substantial scope for improving the discovery power of the machine intelligence framework. For example, incorporation of the 3D structural information of the binding pocket, when available, can be used, together with the sequence information, to encode the target (*51*, *52*), or the encoding of the residues composing the binding pocket can be used to guide the binder molecule design. However, it is noteworthy that guiding the binder generation by the target sequence information opens other avenues of exploration, such as designing of modulators or designing inhibitors that act via different mechanism. For example, the docking and MD simulations indicate that the experimentally validated M^pro^ inhibitors are orthosteric, while the spike inhibitors are allosteric in nature. Specifically, it is established that lipid binding inhibits by an allosteric mechanism, namely, restraining the RBDs to a conformation where they cannot engage the ACE2 receptor. The generations can be further constrained by secondary properties (e.g., solubility, number of hydrogen bonding donor/acceptor sites, and structural diversity), which are potential directions for further work. Iterative optimization methods (*37*), based on experimental feedback, can be adopted to improve the initial discoveries. Active learning paradigms can be explored for improving the process efficiency.

We would like to emphasize that the success of the inhibitor design or subsequent optimization depends on the guidance from, and therefore, accuracy of, the inhibitory potency predictor. The predictor, in the present study, is trained on IC_50_ values reported in the BindingDB database. It is noteworthy that as the drug target becomes dissimilar to the ones covered within the database, as in the case of spike RBD in the present case, the confidence of the AI predictor naturally gets lower. Also, the BindingDB database has a dataset bias toward micromolar level inhibitors, as evident by the mean (6.34) and SD (1.46) of the reported pIC_50_ values, which is carried over to the affinity predictor trained on this database. Further confirming this point, only 14.2% of reported IC_50_ values in our training set are in the range of <0.01 μM, while 81.14% fall in the range of 0.01 to 100 μM.

This proof-of-concept study validates a small number of compounds selected from ≈100 top-ranked AI designs, which are selected based on several factors, such as cost and human effort required for synthesis during a pandemic, as well as off-the-shelf availability of the reactants. It is possible that the compounds that were not selected for final validation can have similar or better inhibitory potential, calling for further large-scale investigation that is beyond the scope this proof-of-concept study. Future work will also consider investigating the effect of training the generative model on broader-scale data, e.g., as used in recently published MoLFormer model (*53*), to explore the previously unexplored areas (molecules with atypical scaffolds) of the inhibitor landscape.

It should be also emphasized that this proof-of-concept study does not include experimental validation of other relevant attributes considered, such as ADME properties, of the discovered molecules, which is beyond the scope of this work. Further studies are needed to confirm binding mechanisms of the proposed inhibitors to SARS-CoV-2 targets. Nevertheless, we believe that, when combined with medicinal chemistry approaches and human expert supervision, the de novo compounds reported here, as well as the framework, may serve as inspiration for a unique and interesting class of broad-spectrum antivirals.

## MATERIALS AND METHODS

### CogMol overview

#### SMILES VAE as a molecule generator

CogMol leverages a VAE (*13*, *54*) paradigm as the base generative model for molecules. The encoder in the VAE encodes molecules to a latent vector representation. The decoder maps latent vectors back to molecules. Arbitrary molecules are generated by sampling from the latent space. Here, molecular SMILES is used as the input and output to the encoder and the decoder, respectively. A bidirectional gated recurrent unit (GRU) with a linear output layer was used as an encoder. The decoder contained a three-layer GRU with a hidden dimension of 512 units and dropout layers with a dropout probability of 0.2. The parameters for the encoder-decoder pair is learned by optimizing a variational lower bound on the log-likelihood of the training data. The loss objective is composed of a reconstruction loss and a Kullback-Leibler (KL) divergence [a measure of divergence between the fixed prior distribution **p**(**z**), standard normal in this case, and the learned distribution **q**_ϕ_(**z**∣**x**)] term:

$$L_{\mathrm{VAE}}(\theta ,\phi )=E_{q_{\phi }(z|x)}[\log p_{\theta }(x|z)]-D_{KL}[q_{\phi }(z|x)\|p(z)]$$

This implies that samples can be generated from random points in the latent space, while points close in the latent space will be decoded into chemically similar molecules.

The VAE was first trained for 40 epochs on 1.6 million chemical molecules from the MOSES benchmarking dataset (*55*), which was chosen from the larger ZINC Clean Leads (*56*) collection. Then, along with the KL and reconstruction loss, the VAE was also jointly trained for another 15 epochs to predict the molecular attributes QED and synthetic accessibility (SA) from the latent vectors **z**. Two separate linear regression models were trained such that the VAE latent space becomes organized based on those physical properties and thus serves as an approximation of the joint probability distribution of molecular structure and the chemical properties (*57*). The training was further continued for 50 epochs on around 211,000 ligand molecules from the BindingDB database (*58*). This paradigm therefore served as a molecule generator that is unbiased toward any particular target. The detailed evaluation of the final model is reported in (*9*).

The final VAE generates SMILES strings by sampling from *q*_ϕ_(**z**∣**x**) that are 99% unique and exhibit greater than 90% chemical validity, while root mean square errors (RMSEs) on the QED and SA prediction are 0.0262 and 0.0175, respectively. The comparison of the unconditionally generated molecules from CogMol with five baseline generative models is reported in table S8, showing comparable performance in terms of producing molecules that are valid, unique, diverse, and pass different medicinal chemistry and other filters.

#### Molecular attribute predictors for conditional generation

Two predictors trained on the latent **z** vectors were used for target-specific inhibitor molecule design, which are also drug-like. The QED regressor was composed of four hidden layers with 50 units each and ReLU nonlinearity. Further, a target-chemical binder (strong/weak) predictor was trained on the latent **z** vectors of chemicals and the pretrained protein sequence embeddings (*15*), which used the data released as part of DeepAffinity (*59*). A pIC_50_ value of >6 was used as a threshold to decide if a compound was a strong binder. The protein embeddings and the molecular embeddings were concatenated and passed through a single hidden layer with 2048 units and ReLU nonlinearity. The **z**-based QED and pIC_50_ predictors yield an RMSE of 0.0281 and 1.282, respectively. These sets of predictors were used for controlled sampling from the VAE model to design molecules with desired attributes.

#### CLaSS sampling used for conditional generation in CogMol

We briefly describe Conditional Latent (attribute) Space Sampling (CLaSS) (*16*) here. CLaSS uses (i) a density model of the VAE latent representation and (ii) a set of molecular attribute predictors trained on the VAE latent vectors to generate molecules in an attribute-controlled manner. For this purpose, a rejection sampling approach using Bayes’ theorem is used. To elaborate further, first an explicit density model is learned on the latent embeddings of the training data to ensure that sampling is uniformly random. A Gaussian mixture model with 100 components and diagonal covariance matrices was used for this purpose. Assuming that the attributes are all independent of each other and can be conditioned on the latent embeddings (i.e., the latent space encompasses all combinations of attributes), Bayes’ rule was then used to define the conditional probability of a sample, given certain properties in terms of the predictor models above. Finally, we use this definition in a rejection sampling scheme such that samples drawn from the density model are accepted according to the product of the attribute predictor scores. For more details, see algorithm S1. Generating the 875,000 samples for each target took around 2 days using an NVIDIA Tesla K80 GPU.

### Ranking and prioritization

The filtering criteria included molecular weight (MW) less than 500 Da, QED greater than 0.5, SA less than 5, and octanol-water partition coefficient (logP) less than 3.5. MW, SA, logP, and QED were calculated using the RDKit toolkit (*60*). A pIC_50_ predictor trained on DeepAffinity (*59*) data was also used for ranking the designed molecules based on predicted affinity (AFF). A SMILES-based binding affinity (pIC_50_) predictor was used for this purpose. SMILES sequences were first embedded using long short-term memory units (LSTMs). Those SMILES embeddings were then concatenated with pretrained protein embeddings (*15*), resulting in RMSE of 0.8426 on the test data. A threshold for predicted pIC_50_ affinity with the respective target sequence was set—greater than 8 for molecules targeting M^pro^ and greater than 7 for molecules targeting the spike RBD. This affinity predictor was also used to estimate target selectivity (SEL) (*9*), defined as the excess affinity to the target compared to a random set of proteins, lack of which is a known cause for drug candidate failure. A positive selectivity value therefore indicates less promiscuous nature of a molecule and is considered as a good candidate for further evaluation.

The molecules were also evaluated for predicted toxicity (*61*) across a total of 12 in vitro (*62*) and 1 clinical end-points (*63*). Morgan fingerprints were used as the input features for the toxicity prediction model. A multitask deep neural network containing a total of four hidden layers was used (*61*): Two layers were shared across all toxicity endpoints, and two were specific to each of the endpoints. A ReLU activation was used for all layers except for the last, for which a sigmoid activation was used. Molecules that were predicted to have no toxicity to any of the toxicity endpoints were progressed in the workflow.

We then ran docking simulations on a prioritized set of designed molecules, less than 1000, with their respective target structures, as the docking energy can provide an indication of actual inhibition. For spike, we used a lipid-bound conformation (PDB ID: 7Z3Z) and kept the protomer frozen during docking, as the goal was to find molecules that dock to the lipid-bound spike RBD. Our intent was to exploit the conserved hydrophobic lipid binding pocket for developing inhibitors that can trap the spike protein in the closed conformation as this is known to have reduced interaction with the host ACE2 receptor (*18*, *19*), rather than targeting the ACE2 binding region that is prone to frequent mutation, resulting in balancing ACE2 receptor binding and allowing escape from neutralizing antibodies. For M^pro^, we used a monomer from the first structure determined and deposited with the PDB for SARS-CoV-2 M^pro^ complexed with the covalent inhibitor N3 [PDB ID: 6LU7 (*31*)] and set the search space to fully encompass the receptor. Docking was performed using AutoDock Vina (*64*) run blindly over the entire protein structure with an exhaustiveness of 8, and repeated five times to find the optimal conformation. Compounds with a binding free energy given by docking of less than −8.4 kcal/mol with M^pro^ were selected. For the generated spike compounds, we prioritized those that exhibited a binding free energy less than −7.5 kcal/mol. Further, we prioritized the compounds that were docked less than 3.9 Å from the lipid binding pocket in the final docked configurations.

The surface and ribbon representations of ligands docked (or bound) to the target structure were produced with either PyMol (*65*) or chimeraX (*66*), and the protein-ligand interaction plots were produced with LigPlot+ (*67*).

In contrast with large-scale screening, docking is only used to provide additional validation of the binding affinity predictor model and therefore can be run after filtering candidates based on the easily computed properties described above. After this filtering, we were left with fewer than 1000 molecules combined between the two targets on which to run docking. Each simulation takes only a few minutes and can be run independently in parallel, which means that the entire in silico screening can be performed in less than a day when run on a compute cluster consisting of Intel Xeon E5-2600 v2 processors.

### Molecular dynamics

MD simulations were started from the lowest energy docked structure. The complexes were then solvated in a box of SPC/E (*68*) water molecules, and two chloride ions were added to neutralize the charge. Simulations were performed at 300 K using periodic boundary conditions and the all-atom optimized potentials for liquid simulations (OPLS-AA) force field (*69*). After equilibrating at proper temperature and pressure, two independent production simulations were run, each for 1 μs, using a modified Berendsen thermostat and a Parrinello-Rahman pressure coupling at 1 atm. GROMACS software (*70*) (version 2022.3) with CUDA support was used for all simulations using an NVIDIA A100 GPU. A 2-fs time step was used. The stability of the complex was monitored using the intermolecular distance as defined in Fig. 5, which was estimated using the gmx distance function. Intermolecular interaction energies from MD simulation were estimated using gmx energy.

### Retrosynthesis prediction

We assessed synthesis plausibility for the previously unidentified compounds, as a major challenge in driving successes in molecular discovery is to devise plausible and efficient synthesis-planning protocols. Here, we applied the recent advances made by machine learning–based approaches to predict retrosynthetic routes from large reaction databases. To estimate the ease of synthesizability and facilitate synthesis planning of the selected compounds, we predicted the retrosynthesis pathways for each candidate using the IBM RXN platform (*17*). RXN combines a transformer neural network for forward reaction prediction and graph exploration techniques to evaluate retrosynthesis paths, scoring them according to probability. The path is terminated when all reagents are found to be commercially available. Candidates for which RXN was unable to determine a feasible retrosynthesis route or which terminated with noncommercially available compounds were removed from consideration. For each prediction, we used the following parameters: maximum single step reactions (depth), 6; minimum acceptance probability for a single step, 0.6; maximum number of pathways (beams), 10; number of steps between removal of low probability steps (pruning), 2; and maximum execution time, 1 hour. Commercial availability was determined by searching the eMolecules database (*71*) with a restriction on lead time of 4 weeks or less but no restriction on price.

In the next section, we provide a detailed comparisons between predicted retrosynthesis and actual synthesis routes, which is also summarized in table S4. We considered three main aspects in the comparison: number of reaction steps leading to the final product, overlap of the products in the intermediate reaction steps, and overlap of reactants used in the reactions. We chose the best path from the top six predicted for comparison by optimizing first for product overlap and then for reactant overlap. Overall, the total number of actual reaction steps showed good agreement with predictions, generally only off by one or two steps. This was confirmed by the overlap of intermediate products, which showed that retrosynthesis often predicted the correct high-level path. Product overlap is highly variable, though, since there are relatively few per route (often only two or three). The actual synthesis routes even used many of the same reactants as predicted, although occasionally alternatives had to be found due to stock limitations. In general, the retrosynthesis prediction was used as a starting point and any “major” deviations required were considered a failure. Around 90 to 95% of the top 100 generated compounds turned out to be synthesizable, based on the retrosynthesis pathway predictions by IBM RXN (*9*) and human evaluation from SMEs at Enamine. Design prioritization to a small representative set was achieved by considering time, reactant and reagent availability, and amount of human effort.

### Synthesis protocols

In this section, we compare the retrosynthesis predictions to the actual routes used to synthesize the molecules: GEN727 was synthesized according to the best RXN-predicted method (fig. S1). The synthesis of GEN725 was carried out by analogy to the best RXN strategy. SNAr ester synthesis in *N*,*N*′-dimethylformamide (DMF) gave intermediate compound **13** with high yield. Cross-coupling of **13** with sulfonamide-pinacolborane led to the final product with a moderate yield (fig. S2). Several unsuccessful attempts were made to carry out the first step according to the retrosynthetic strategy for GEN626, which led to obtaining the desired intermediate with very low yield. As a result, the synthetic pathway was changed. SNAr reaction was carried out with cyanide **8**, which was followed by hydrolysis of intermediate compound **10** (obtained with a moderate yield). Reduction of nitro-group of **11** led to GEN626 (fig. S3). Unfortunately, following the pathway suggested by retrosynthesis for GEN777 did not give good results and the synthetic strategy needed to be changed. We synthesized acyl chloride **5**, which reacted with methyl amine on the next step. Thereafter, amide **6** was treated by PCl_5_ and the resulting intermediate was reacted in situ with azide anion (fig. S4).

Synthesis orders for designed compounds were placed with Enamine on 4 August 2020 (received by Enamine PO:8000109) and on 4 September 2020 (received by Enamine PO:8001023). Structures were added to the automated chemical design (ACD) commercial database as a part of regular auto-update of Enamine’s catalog. Enamine did not have boc-amino pinacolborane **20** in stock and could not follow the proposed retrosynthetic strategy for GXA104. Unprotected amino-pinacolborane was available, and so the strategy was changed, which made it possible to obtain GXA104 in fewer steps. At first, **20** was reacted with carboxylic acid **19**, which led to amide **21**. Cross-coupling of **21** with 3-iodo-1*H*-indazole led to GXA104 (fig. S5). GXA56 was synthesized according to the top RXN-predicted method (fig. S6). GXA70 was synthesized by analogy to the best RXN-predicted method. Minor modifications were made to the synthetic steps, such as use of other bases and organic solvents (not notable for a whole scheme). The RXN strategy was chosen due to high reactivity of trichlorotriazine with amines and the need to substitute only one chlorine at the first stage (it is easier to be controlled with less nucleophilic aniline compared to more nucleophilic aliphatic secondary amines) (fig. S7). The RXN-predicted strategy for GXA112 was followed as closely as possible. The last synthetic step [reaction with SO_2_(NH_2_)_2_] led to the final product with very low yield. To improve it, mono-Boc–protected SO_2_(NH_2_)_2_ was synthesized and reacted with **26**. Boc-protected final product **30** was obtained and readily deprotected via trifluoroacetic acid cocktail (fig. S8). Spectroscopic characterization of synthesized de novo compounds can be found in table S9.

### Cloning, protein production, and crystallization

#### M^pro^ production

The M^pro^ coding sequence was codon-optimized for expression in *Escherichia coli* and synthesized by Integrated DNA Technologies. The M^pro^ expression construct used for crystallization comprises an N-terminal glutathione *S*-transferase region, an M^pro^ autocleavage site, the M^pro^ coding sequence, a hybrid cleavage site recognizable by 3C Human Rhinovirus (HRV) protease, and a C-terminal 6-histidine tag (*72*). The overall construct was flanked by In-Fusion compatible ends for insertion into Bam HI–Xho I cleaved pGEX-6P-1 (Sigma). Protein expression, purification, and crystallization were carried out in similar conditions to those previously described by Douangamath *et al.* (*73*). Specifically, crystals were obtained from 0.1 M MES (pH 6.5), 15 PEG4K, and 5% dimethyl sulfoxide (DMSO) using drop ratios of 0.15 μl of protein, 0.3 μl of reservoir solution, and 0.05 μl of seed stock.

#### Genetic constructs of spike ectodomain

The construct is comprised of the gene encoding amino acids 1 to 1208 of the SARS-CoV-2 spike glycoprotein ectodomain, with mutations of RRAR > GSAS at residues 682 to 685 (the furin cleavage site) and KV > PP at residues 986 to 987, as well as inclusion of a T4 fibritin trimerization domain, an HRV 3C cleavage site, a 8xHis tag and a Twin-Strep-tag at the C terminus, as reported by Wrapp *et al.* (*74*). All vectors were sequenced to confirm that clones were correct.

#### Spike protein production

Recombinant spike ectodomain was expressed by transient transfection in human embryonic kidney (HEK) 293S GnTI- cells (American Type Culture Collection, CRL-3022) for 9 days at 30°C. Conditioned medium was dialyzed against 2× phosphate-buffered saline (pH 7.4) buffer. The spike ectodomain was purified by immobilized metal affinity chromatography using Talon resin (Takara Bio) charged with cobalt followed by size exclusion chromatography using HiLoad 16/60 Superdex 200 column in 150 mM NaCl, 10 mM Hepes (pH 8.0), 0.02% NaN_3_ at 4°C.

### X-ray screening of M^pro^ binding compounds

Compounds were dissolved in DMSO and directly added to the crystallization drops, giving a final compound concentration of 10 mM and DMSO concentration of 10%. The crystals were left to soak in the presence of the compounds for 1 to 2 hours before being harvested and flash-cooled in liquid nitrogen without the addition of further cryoprotectant. X-ray diffraction data were collected on beamline I04-1 at Diamond Light Source and automatically processed using the Diamond automated processing pipelines (*75*). Analysis was performed as outlined previously (*73*). Briefly, XChemExplorer (*76*) was used to analyze each processed dataset that was automatically selected, and electron density maps were generated with Dimple (*77*). Ligand binding events were identified using PanDDA (*78*), and ligands were modeled into PanDDA-calculated event maps using Coot (*79*). Restraints were calculated with AceDRG (*80*) or GRADE (*81*), structures were refined with Refmac (*82*) and Buster (*83*), and models and quality annotations were cross-reviewed. We have added PanDDA event maps in fig. S19 for structures of the protein-hit complexes obtained. The PanDDA algorithm takes advantage of the large number of datasets collected during a fragment campaign to detect partial-occupancy ligands that are typically not readily detected in normal crystallographic maps and thus provides a better indication of bound compounds or hits than traditional omit maps.

### Dose response assay for measuring M^pro^ inhibition

The solid-phase extraction C4-cartridge coupled RapidFire 365 Mass Spectrometry (SPE RFMS)–based high-throughput dose response assay has been described (*25*). In brief, M^pro^ inhibitors were dry dispensed in an 11-point threefold dilution series using acoustic liquid transfer robot (Labcyte 550) in 384-well polypropylene plate (Greiner Bio-One). M^pro^ (0.3 μM) was dispensed across the well (25 μl per well) using Multidrop Combi (Thermo Scientific), and the reaction was incubated at ambient temperature. Compounds were incubated with the protein for 15 min, following which the 11-mer substrate peptide TSAVLQ/SGFRK-NH_2_ (4 μM) was dispensed (25 μl per well) for probing inhibition activity. Reaction was quenched by addition of 10% aqueous formic acid (5 μl per well) after 10-min incubation with the substrate at an ambient temperature. After addition of each reagent, the plates were centrifuged for 30 s (Axygen Plate Spinner Centrifuge). Samples were analyzed by RapidFire (RF) 365 high-throughput sampling robot (Agilent) connected to an iFunnel Agilent 6550 accurate mass quadrupole time-of-flight (Q-TOF) mass spectrometer [operating parameters: capillary voltage (4000 V), nozzle voltage (1000 V), fragmentor voltage (365 V), drying gas temperature (280°C), gas flow (13 liters/min), sheath gas temperature (350°C), sheath gas flow (12 liters/min)]. The peptide/protein sample was loaded onto a solid-phase extraction (SPE) C4-cartridge and washed with 0.1% (v/v) aqueous formic acid to remove nonvolatile buffer salts (5.5 s, 1.5 ml/min) before elution with aqueous 85% (v/v) acetonitrile containing 0.1% (v/v) formic acid (5.5 s, 1.25 ml/min). The cartridge was reequilibrated with 0.1% (v/v) aqueous formic acid (0.5 s, 1.25 ml/min), and sample aspirator was washed with an organic and aqueous wash before the injection of next protein: peptide mixture sample onto the SPE cartridge.

Data were extracted with RapidFire integrator software (Agilent), and mass/charge ratio (*m*/*z*) (+1) was used for both N-terminal fragment TSAVLQ (681.34 Da) and the 11-mer substrate peptide (1191.68 Da). The percentage M^pro^ activity [N-terminal product peak integral/(N-terminal product peak integral + substrate peak integral) ∗ 100] was calculated in Microsoft Excel, and normalized data were transferred to Prism 9 for nonlinear regression curve analysis. IC_50_ values are reported as the mean of technical duplicates (*n* = 2; mean ± SD). Signal to noise and *Z*′ factor were calculated in Microsoft Excel (*Z*′ > 0.8) (*25*).

### Spike thermal shift-based binding assay

Thermofluor (differential scanning fluorimetry) experiments were performed in triplicate in 96-well white polymerase chain reaction (PCR) plates using a 1300-fold excess of small molecule (in DMSO) to 1.5 μg of spike monomer in 50 μl of buffer per well. An Agilent MX3005p RT-PCR instrument (λ_ex_ 492 nm/λ_em_ 585 nm) was used to monitor the fluorescence change of a 3× final concentration of SYPRO Orange dye (Thermo) in an “increasing-sawtooth” temperature profile where the temperature was increased in 1°C increments from 25°C to 98°C with the fluorescence recorded at 25°C. Four of the synthesized compounds were investigated using thermofluor assay to assess effect upon stability. Several conditions were tested: in 20 mM sodium acetate (pH 4.6), 150 mM NaCl, a storage buffer at which long term stability was observed to be much improved (*84*); in 50 mM Hepes (pH 7.5), 200 mM NaCl immediately after buffer exchange from the storage buffer; after incubation overnight at pH 7.5; and after incubation overnight at pH 7.5 in the presence of the compound. Raw fluorescence data were analyzed using Microsoft Excel and the JTSA software (*85*) using a five-parameter model to produce melting temperature (*T*_m_) values. Note that fresh spike protein exhibits a single melting transition, which can be characterized as a melting point, *T*_m_, of 65°C in neutral pH buffer. At a reduced pH 4.6, the single melting transition is at 62°C. As spike is incubated in pH 7.5, a second transition appears at a lower temperature with a *T*_m_ of 50°C. This transition increases as a proportion of the total melt until it is the only transition observed and correlates with a presumed conformational change of the spike trimer to a less stable form.

### Focus reduction neutralization assay for measuring SARS-CoV-2 live virus neutralization of spike RBD-targeting compounds

Vero-CCL-81 cells (100,000 cells per well) were seeded in a 96-well, cell culture–treated, flat-bottom microplates for 48 hours. Compounds were serially diluted and incubated with approximately 100 foci of SARS-CoV-2 for 1 hour at 37°C. The mixtures were added on cells and incubated for further 2 hours at 37°C followed by the addition of 1.5% semisolid carboxymethyl cellulose (CMC) overlay medium to each well to limit virus diffusion. Twenty hours after infection, cells were fixed and permeabilized with 4% paraformaldehyde and 2% Triton X-100, respectively. The virus foci were stained with human anti-SARS-CoV-2 nucleocapsid protein (anti-NP) mAb (mAb206) and peroxidase-conjugated goat anti-human immunoglobulin G (IgG) (A0170; Sigma), and visualized by adding Trueblack Peroxidase Substrate. Virus-infected cell foci were counted on the classic AID EliSpot reader using AID ELISpot software. The percentage of focus reduction was calculated by comparing the number of foci in treated wells with the number in untreated control wells, and IC_50_ was determined using the probit program from the SPSS package.

### Pseudoviral neutralization assay for measuring inhibition of SARS-CoV-2 pseudovirus entry of spike RBD-targeting compounds

Pseudotyped lentiviral particles expressing SARS-CoV-2 S protein were incubated with serial dilutions of compounds in white opaque 96-well plates for 1 hour at 37°C. The stable HEK293T/17 cells expressing human ACE2 were then added to the mixture at 15,000 cells per well. Plates were spun at 500 relative centrifugal force (RCF) for 1 min and further incubated for 48 hours. Finally, culture supernatants were removed followed by the addition of Bright-Glo Luciferase assay system (Promega, USA). The reaction was incubated at room temperature for 5 min, and the firefly luciferase activity was measured using CLARIOstar (BMG Labtech). The percentage of neutralization of compounds toward pseudotyped lentiviruses was calculated relative to the untreated control, and IC_50_ was determined using the probit program from the SPSS package.

## Supplementary Materials

### This PDF file includes:

Tables S1 to S9 Figs. S1 to S19 Supplementary Text Legends for 5SML PanDDA event files Legends for 5SMM PanDDA event files Legends for 5SMN PanDDA event files Legend for supplementary code Legend for COVID-19 Molecule Explorer (Mpro) Legend for COVID-19 Molecule Explorer (RBD) References

### Other Supplementary Material for this manuscript includes the following:

5SML PanDDA event files

5SMM PanDDA event files

5SMN PanDDA event files

Supplementary code

COVID-19 Molecule Explorer (Mpro)

COVID-19 Molecule Explorer (RBD)

## Notes

View/request a protocol for this paper from *Bio-protocol*.
