# Supplementary material for: Accelerating drug target inhibitor discovery with a deep generative foundation model
Source: Sci Adv. 2023 Jun 21;9(25):eadg7865. doi: 10.1126/sciadv.adg7865 (PMC10284550; doi:10.1126/sciadv.adg7865)
Supplement: Supplementary file 1 — Tables S1 to S9 Figs. S1 to S19 Supplementary Text Legends for 5SML PanDDA event files Legends for 5SMM PanDDA event files Legends for 5SMN PanDDA event files Legend for supplementary code Legend for COVID-19 Molecule Explorer (Mpro) Legend for COVID-19 Molecule Explorer (RBD) References [file sciadv.adg7865_sm.pdf]

Science Advances
AAAS

Supplementary Materials for

Accelerating drug target inhibitor discovery with a deep generative foundation model

Vijil Chenthamarakshan *et al.*

Corresponding author: Martin A. Walsh, martin.walsh@diamond.ac.uk; David I. Stuart, david.stuart@strubi.ox.ac.uk; Payel Das, daspa@us.ibm.com

Sci. Adv. **9**, eadg7865 (2023)
DOI: 10.1126/sciadv.adg7865

The PDF file includes:

Tables S1 to S9
Figs. S1 to S19
Supplementary Text
Legends for 5SML PanDDA event files
Legends for 5SMM PanDDA event files
Legends for 5SMN PanDDA event files
Legend for supplementary code
Legend for COVID-19 Molecule Explorer (Mpro)
Legend for COVID-19 Molecule Explorer (RBD)
References

Other Supplementary Material for this manuscript includes the following:

5SML PanDDA event files
5SMM PanDDA event files
5SMN PanDDA event files
Supplementary code
COVID-19 Molecule Explorer (Mpro)
COVID-19 Molecule Explorer (RBD)

Supplementary Materials

Supplementary Tables

Table S1. SARS-CoV-2 target protein sequences. The amino acid sequences of the protein targets used in the generation pipeline

Target	Sequence
M^{pro}	SGFRKMAFPSGKVEGCMVQVTCGTTTLNGLWLDDVVYCPRHVICTSEDMLNPNYEDLLIRKSNHNFL<br>VQAGNVQLRVIGHSMQNCVLKLKVDTANPKTPKYKFVRIQPGQTFSVLACYNGSPSGVYQCAMRPNF<br>TIKGSFLNGSCGSVGFNIDYDCVSFCYMHHMELPTGVHAGTDLEGNFYGPFVDRQTAQAAGTDTTIT<br>VNVLAWLYAAVINGDRWFLNRFTTTLNDFNLVAMKYNYEPLTQDHVDILGPLSAQTGIAVLDMCASL<br>KELLQNGMNGRTILGSALLEDEFTPFDVVRQCSGVTFQ
Chimeric RBD	RVVPSGDVVRFPNITNLCPFGEVFNATKFPSVYAWERKKISNCVADYSVLYNSTFFSTFKCYGVSAT<br>KLNDLCFSNVYADSFVVKGDDVRQIAPGQTGVIADYNYKLPDDFMGCVLAWNTRNIDATSTGNYNYK<br>YRLFRKSNLKPFERDISTEIYQAGSTPCNGVEGFNCYFPLQSYGFQPTNGVGYQPYRVVVLSFELLN<br>APATVCGPKLSTDLIK

Table S2. Predicted and estimated properties of *de novo* compounds targeting M^{pro}. See the Ranking and prioritization section for explanations of the column headers.

ID	AFF (pIC_{50})	SEL (pIC_{50})	TOX	QED	SA	logP	MW (Da)	docking (kcal/mol)	dist. to pocket (Å)
GXA56	8.050	0.646	0	0.695	2.562	3.337	404.305	−9.2	3.88
GXA70	8.162	0.744	0	0.771	2.774	3.301	430.503	−9.1	6.77
GXA104	8.16	1.112	0	0.730	2.417	3.484	376.460	−8.9	6.65
GXA112	8.280	0.721	0	0.610	2.934	0.943	488.618	−8.8	4.97

Table S3. Predicted and estimated properties of *de novo* compounds targeting spike RBD. See the Ranking and prioritization section for explanations of the column headers.

ID	AFF (pIC_{50})	SEL (pIC_{50})	TOX	QED	SA	logP	MW (Da)	docking (kcal/mol)	dist. to pocket (Å)
GEN626	7.077	0.754	0	0.829	2.392	1.773	317.311	−7.6	1.93
GEN725	8.140	0.752	0	0.704	1.951	3.197	403.481	−8.8	2.06
GEN727	7.920	0.826	0	0.857	2.322	3.382	293.414	−8.1	2.69
GEN777	7.513	0.834	0	0.819	2.603	2.333	248.717	−7.9	3.36

Table S4. Consolidated results comparing predicted and actual synthesis paths. The top 6 predicted retrosynthesis paths (by confidence) are considered and the path with the best agreement is shown. “Steps” is simply the number of reaction steps actual / predicted number of reaction steps. “Products” shows the intermediate (not including the final molecule) reaction products overlap in terms of recall (with respect to the predicted path) while “reactants” similarly shows the overlap of reactants from all steps in terms of recall. The “success” column shows whether the given predicted path was successfully synthesized as is or with minor changes or failed (but still synthesized via an alternative method devised by Enamine).

		path	conf.	steps	products	reactants	success	comments
GEN626	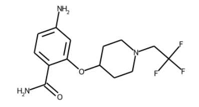	5	1.0	150%	50.0%	62.5%	✗	
GEN725	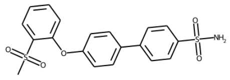	5	1.0	66.7%	50.0%	50.0%	✓	minor changes; moderate yield
GEN727	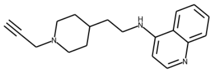	5	1.0	100%	100%	70.0%	✓	followed top prediction
GEN777	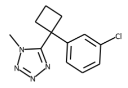	3	1.0	200%	33.3%	75.0%	✗	
GXA56	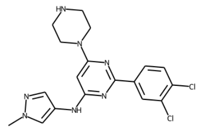	0	1.0	100%	100%	52.9%	✓	followed top prediction
GXA70	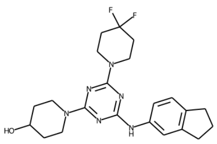	2	1.0	100%	100%	38.5%	✓	minor changes to top prediction
GXA104	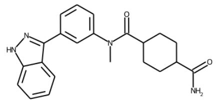	0	0.88	66.7%	0%	35.7%	–	reactant unavailable
GXA112	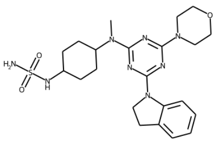	4	1.0	140%	75.0%	62.5%	✓	low yield

Table S5. Crystallographic data collection and refinement statistics. Values in parentheses refer to the highest resolution shell.

	Z68337194<br>5SML	Z1633315555<br>5SMM	Z1365651030<br>5SMN
Data Collection			
Wavelength (Å)	0.9126	0.9126	0.9126
Resolution range (Å)	47.57-1.53 (1.585-1.53)	47.8-1.58 (1.64-1.58)	47.36-1.36 (1.41-1.36)
Space group	C2	C2	C2
Unit cell			
a,b,c (Å)	112.12, 52.83, 44.46	113.12, 53.04, 44.38	111.93, 52.57, 44.59
α,β,γ (°)	90.00, 102.99, 90.00	90.00, 102.90, 90.00	90.00, 102.94, 90.00
Total reflections	119085 (10469)	112151 (10502)	158637 (10806)
Unique reflections	38187 (3690)	35130 (3385)	53606 (4976)
Multiplicity	3.1 (2.7)	3.2 (3.0)	3.0 (2.1)
Completeness (%)	98.88 (95.80)	99.16 (96.18)	98.60 (92.03)
Mean $I/\sigma I$	11.16 (0.81)	12.16 (0.76)	14.56 (0.77)
R_{merge}	0.088 (0.937)	0.097 (1.38)	0.068 (1.02)
R_{meas}	0.106 (1.164)	0.117 (1.68)	0.082 (1.32)
CC1/2	0.995 (0.342)	0.997 (0.336)	0.998 (0.347)
Refinement			
Reflections used in refinement	37923 (3674)	34946 (3378)	53514 (4976)
R_{work}	0.1962 (0.3414)	0.1966 (0.3680)	0.1934 (0.3734)
R_{free}	0.2250 (0.3324)	0.2322 (0.3891)	0.2181 (0.3577)
Number of non-hydrogen atoms	4084	3818	3304
Protein	3598	3412	2935
Ligands	54	76	54
Solvent	432	330	315
RMSD bond lengths (Å)	0.013	0.013	0.014
RMSD bond angles (°)	1.73	1.77	1.81
Ramachandran favored (%)	97.35	97.68	97.68
Ramachandran allowed (%)	2.32	1.99	1.99
Ramachandran outliers (%)	0.33	0.33	0.33
Rotamer outliers (%)	1.00	2.08	0.31
Clashscore	5.4	3.91	3.74
Average B-factors (Å^2)			
All	23.19	23.55	18.92
Protein	22.21	22.55	17.82
Solvent	31.33	32.25	27.53

Table S6. Molecular similarity with existing inhibitors. Tanimoto similarity of the validated candidate hits (columns) to existing SARS-CoV-2 M^{pro} inhibitors (rows). We considered the following inhibitors for comparison: an aminipyridine hit identified in the COVID-19 Moonshot initiative[86], X77 identified using ultralarge docking[38], the oral inhibitor S-217622 from reference[87] Nirmatrelvir in PAXLOVID[32], an α-ketoamide inhibitor (Compound 21 from Zhang, et al.[28]), and Molnupiravir[88]. Consistently, the CogMol-designed inhibitors show high dissimilarity (as indicated by a low Tanimoto similarity around 0.1) to existing SARS-CoV-2 M^{pro} inhibitors.

		GXA70 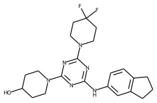	GXA112 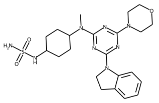	Z68337194 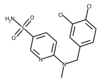
TRY-UNI-714a760b-6[86]	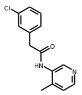	0.101	0.091	0.200
X77[38]	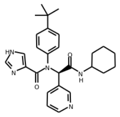	0.116	0.150	0.115
Ensitrelvir (S-217622)[87]	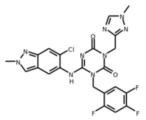	0.093	0.075	0.128
Nirmatrelvir (PF-07321332)[32]	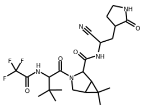	0.109	0.100	0.051
Compound 21[28]	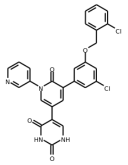	0.077	0.080	0.132
Molnupiravir[88]	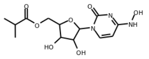	0.146	0.170	0.118

Table S7. ADME properties of validated hits. Drug-likeness (as estimated using number of violations according to Lipinski's[89], Ghose's[90], Veber's[91], Egan's[92], and Muegge's[93] criteria), bioavailability[94] (Low below 0.25, Medium between 0.25 and 0.75, and High above 0.75), number of medicinal chemistry (PAINS[95] and BRENK[96]) alerts and Leadlikeness[97] (number of violations: 250 g/mol $\leq$ molecular weight $\leq$ 400 g/mol, xlogP $\leq$ 3.5, number of rotatable bonds $\leq$ 7) are estimated using SwissADME software[36].

ID	Lipinski	Ghose	Veber	Egan	Muegge	Bioavailability	PAINS	BRENK	Leadlikeness
Z68337194	0	0	0	0	0	Medium	0	0	0
GXA70	0	0	0	0	0	Medium	0	0	2
GXA56	0	0	0	0	0	Medium	0	0	1
GEN725	0	0	0	0	0	Medium	0	0	1
GEN727	0	0	0	0	0	Medium	0	1	0

Table S8. Comparison of generated molecules in terms of fraction of valid, unique (out of 1,000 and 10,000 generated), internal diversity, and passing filters (medicinal chemistry filters, PAINS, ring sizes, charges, atom type). All generative models were trained and tested on MOSES benchmark[55]. Performances of baseline models are from Polykovskiy, et al.[55].

Model	Valid	Unique@1k	Unique@10k	IntDiv1	IntDiv2	Filters
CogMol[9]	0.95	1.0	0.999	0.8578	0.8521	0.9888
CharRNN[98]	0.809	1.0	1.0	0.855	0.849	0.975
AAE[99]	0.997	1.0	0.995	0.857	0.85	0.997
VAE[57]	0.969	1.0	0.999	0.856	0.851	0.996
JT-VAE[100]	1.0	1.0	0.999	0.851	0.845	0.978
LatentGan[101]	0.8966	1.0	0.9968	0.8565	0.8505	0.9735
Training	1.0	1.0	1.0	0.857	0.851	1.0

Table S9. Compound characterization. Nuclear magnetic resonance (NMR) and high pressure liquid chromatography-mass spectrometry (HPLC-MS).

GEN727	^{1}H NMR (400 MHz, dmso) δ 8.37 (d, J = 5.3 Hz, 1H), 8.21 (d, J = 8.4 Hz, 1H), 7.76 (d, J = 8.4 Hz, 1H), 7.59 (t, J = 7.6, 7.6 Hz, 1H), 7.40 (t, J = 7.6, 7.6 Hz, 1H), 7.08 (t, J = 5.4, 5.4 Hz, 1H), 6.42 (d, J = 5.3 Hz, 1H), 3.29 (q, J = 6.7, 6.7, 6.4 Hz, 2H), 3.22 (d, J = 2.5 Hz, 2H), 3.10 (t, J = 2.5, 2.5 Hz, 1H), 2.76 (dt, J = 11.8, 3.4, 3.4 Hz, 2H), 2.08 (td, J = 11.5, 11.4, 2.6 Hz, 2H), 1.72 (m, 2H), 1.60 (q, J = 7.1, 7.1, 7.1 Hz, 2H), 1.37 (m, 1H), 1.20 (qd, J = 12.0, 11.8, 11.8, 3.8 Hz, 2H).<br>HPLC-MS m/z [M+H]+ = 294.2 , purity 100%
GEN777	^{1}H NMR (400 MHz, dmso) δ 7.41 (m, 1H), 7.37 (m, 2H), 7.19 (d, J = 7.5 Hz, 1H), 3.64 (s, 3H), 2.94 (m, 2H), 2.75 (m, 2H), 1.98 (m, 2H).<br>HPLC-MS m/z [M+H]+ = 249.2 , purity 100%
GEN626	^{1}H NMR (400 MHz, dmso) δ 7.63 (d, J = 8.6 Hz, 1H), 7.28 (br s, 1H), 7.08 (br s, 1H), 6.28 (d, J = 2.0 Hz, 1H), 6.18 (dd, J = 8.5, 2.0 Hz, 1H), 5.65 (m, 2H), 4.42 (m, 1H), 3.20 (q, J = 10.3, 10.2, 10.2 Hz, 2H), 2.83 (m, 2H), 2.60 (m, 2H), 1.98 (m, 2H), 1.74 (m, 2H).<br>HPLC-MS m/z [M+H]+ = 318.2 , purity 100%
GEN725	^{1}H NMR (400 MHz, dmso) δ 7.97 (dd, J = 7.9, 1.8 Hz, 1H), 7.90 (m, 4H), 7.82 (d, J = 8.8 Hz, 2H), 7.73 (td, J = 7.9, 7.8, 1.8 Hz, 1H), 7.41 (m, 3H), 7.26 (d, J = 8.7 Hz, 2H), 7.14 (d, J = 8.3 Hz, 1H), 3.38 (s, 3H).<br>HPLC-MS m/z [M+H]+ = 404.2 , purity 98.72%
GXA104	^{1}H NMR (400 MHz, dmso) δ 13.34 (s, 1H), 8.08 (d, J = 8.3 Hz, 1H), 8.01 (d, J = 8.3 Hz, 1H), 7.87 (s, 1H), 7.61 (d, J = 8.3 Hz, 2H), 7.42 (m, 1H), 7.36 (d, J = 7.7 Hz, 1H), 7.23 (m, 1H), 7.11 (br s, 1H), 6.59 (br s, 1H), 3.22 (s, 3H), 2.23 (m, 1H), 2.01 (m, 1H), 1.69 (m, 4H), 1.42 (m, 2H), 1.00 (m, 2H).<br>HPLC-MS m/z [M+H]+ = 377.2 , purity 100%
GXA112	^{1}H NMR (500 MHz, dmso) δ 8.18 (s, 1H), 7.18 (d, J = 7.1 Hz, 1H), 7.13 (t, J = 7.7, 7.7 Hz, 1H), 6.86 (m, 1H), 6.64 (m, 1H), 6.46 (m, 2H), 4.44 (m, 1H), 4.09 (m, 2H), 3.69 (m, 4H), 3.62 (m, 4H), 3.50 (m, 1H), 3.07 (m, 2H), 2.98 (m, 2H), 2.94 (m, 1H), 2.07 (m, 1H), 1.90 (m, 2H), 1.67 (m, 1H), 1.59 (m, 2H), 1.36 (m, 2H).<br>HPLC-MS m/z [M+H]+ = 489.2 , purity 100%
GXA70	^{1}H NMR (400 MHz, dmso) δ 8.96 (s, 1H), 7.53 (s, 1H), 7.40 (dd, J = 8.0, 2.1 Hz, 1H), 7.08 (d, J = 8.1 Hz, 1H), 4.71 (d, J = 4.2 Hz, 1H), 4.23 (m, 2H), 3.84 (m, 4H), 3.71 (m, 1H), 3.22 (m, 2H), 2.79 (m, 4H), 1.97 (m, 6H), 1.75 (m, 2H), 1.32 (m, 2H).<br>HPLC-MS m/z [M+H]+ = 431.2 , purity 100%
GXA56	^{1}H NMR (400 MHz, dmso) δ 8.96 (s, 1H), 7.53 (s, 1H), 7.40 (dd, J = 8.0, 2.1 Hz, 1H), 7.08 (d, J = 8.1 Hz, 1H), 4.71 (d, J = 4.2 Hz, 1H), 4.23 (m, 2H), 3.84 (m, 4H), 3.71 (m, 1H), 3.22 (m, 2H), 2.79 (m, 4H), 1.97 (m, 6H), 1.75 (m, 2H), 1.48 (m, 2H).<br>HPLC-MS m/z [M+H]+ = 404.2 , purity 100%

Supplementary Figures

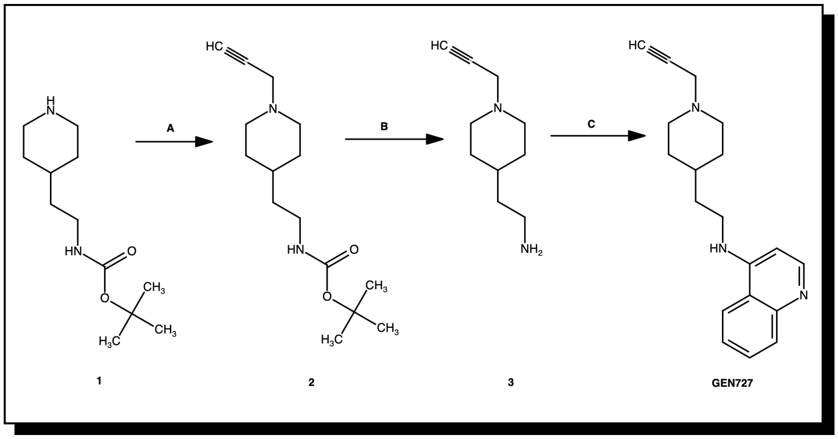

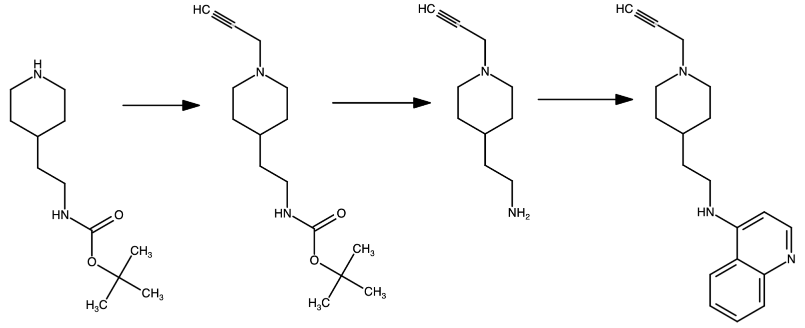

Fig. S1. GEN727 synthesis route (top) and RXN-predicted retrosynthetic pathway (bottom). **(A)** A mixture of compound **1** (0.5 g, 2.2 mmol), propargyl bromide (0.4 g, 3.3 mmol) and potassium carbonate (0.6 g, 4.4 mmol) was suspended in acetonitrile (20 mL) and the reaction mixture was heated to 60 °C for 18 h. The solids were removed via filtration and the solvent was removed *in vacuo*. The residue was diluted with an aqueous $NaHSO_4$ solution (50 mL) and washed with dichloromethane (2 × 20 mL); the aqueous layer was basified with NaOH to pH=14, and extracted with dichloromethane (3 × 30 mL). The organic extracts were combined, dried over Na_2SO_4 and concentrated *in vacuo* to obtain crude **2** (0.4 g) which was used in the next step without purification. **(B)** Crude compound **2** (0.4 g) was dissolved in methanol (10 mL) and a hydrogen chloride solution in dioxane (20 mL) was added. The reaction mixture was stirred for 18 h at 20 °C. The volatiles were removed *in vacuo* to obtain crude **3** (0.32 g) as a hydrochloride salt. **(C)** Crude compound **3** (0.32 g) was dissolved in DMSO (5 mL), 4-chloroquinoline (0.330 g, 2 mmol) and DIPEA (0.65 g, 5 mmol) were added to the solution. The reaction mixture was stirred at 100 °C for 48 h and purified via preparative HPLC to obtain **GEN727** (2 fractions: 0.0257 g and 0.0278 g, overall yield 9%) as brown solid.

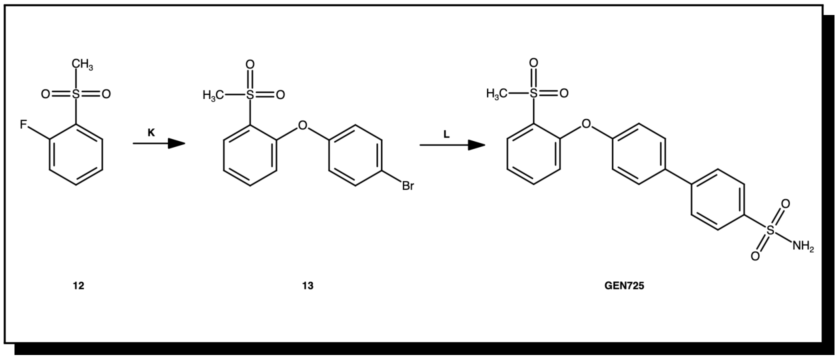

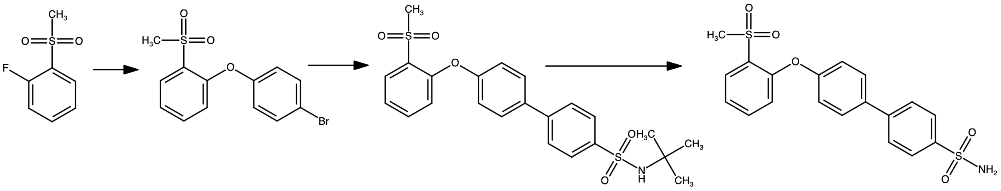

Fig. S2. GEN725 synthesis route (top) and RXN-predicted retrosynthetic pathway (bottom). (K) To a suspension of NaH (0.250 g, 6.31 mmol, 60% dispersion in mineral oil) in DMF (5 mL) was added dropwise a solution of 4-bromophenol (1.09 g, 6.31 mmol) in DMF (5 mL). The mixture was stirred for 1 h and compound **12** (1 g, 5.74 mmol) was added. The reaction mixture was stirred at 100 °C overnight, cooled to r.t. and poured into ice (100 mL). The precipitate was filtered and washed with water (3 × 10 mL) and with hexanes. The solid was dried *in vacuo* to give **13** (1.72 g, 92%). **(L)** To a mixture of compound **13** (1 g, 3.06 mmol), 4-(4,4,5,5-tetramethyl-1,3,2-dioxaborolan-2-yl)-benzenesulfonamide (1.04 g, 3.67 mmol) and sodium carbonate (0.81 g, 7.65 mmol) in a mixture of dioxane and water (9:1, 10 mL) was added XPhos Pd G3 (0.260 g, 0.36 mmol) under an inert atmosphere. The reaction mixture was stirred for 16 h at 95 °C (oil bath), cooled to r.t., diluted with water (10 mL) and extracted with EtOAc (2 × 10 mL). The combined organic layers were dried over Na_2SO_4 and evaporated under reduced pressure. The residue was purified by preparative HPLC to give **GEN725** (0.304 g, 25%).

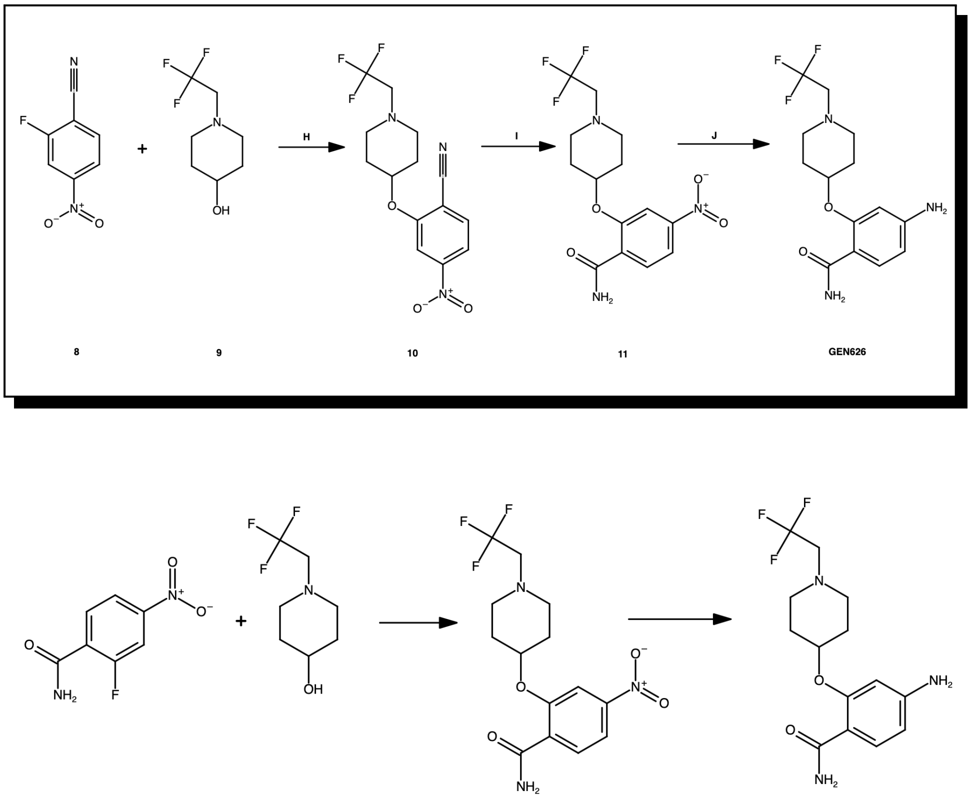

Fig. S3. GEN626 synthesis route (top) and RXN-predicted retrosynthetic pathway (bottom). (H) To a solution of compound **9** (0.55 g, 3 mmol) in dry DMF (15 mL), sodium hydride (as 60% suspension in mineral oil, 0.132 g, 3.3 mmol) was added in one portion. The mixture was stirred at 40 °C for 30 min and compound **8** (0.5 g, 3 mmol) was added. The reaction mixture was stirred at 20 °C for 18 h, diluted with water (100 mL), and extracted with ethyl acetate (3 × 30 mL). The combined organic layers were washed with water (4 × 50 mL), dried over Na_2SO_4 and concentrated *in vacuo* to obtain the crude material which was purified via column chromatography ($CHCl_3$:MeOH 10:1 as eluent) to afford **10** (0.18 g, 0.55 mmol, 18% yield) as yellow oil. **(I)** Compound **10** (0.18 g, 0.55 mmol) was suspended in conc. H_2SO_4 (5 mL) and the reaction mixture was heated to 60 °C for 2 h, cooled with ice and diluted with an aqueous Na_2CO_3 solution to basic pH. The resulting mixture was extracted with ethyl acetate (3 × 30 mL); the organic layer was dried over Na_2SO_4 and concentrated *in vacuo* to obtain **11** (0.16 g, 0.46 mmol, 84% yield) as yellow solid. **(J)** To a solution of compound **11** (0.16 g, 0.46 mmol) in methanol (10 mL), Pd/C (10%w, 0.100 g) was added. The reaction mixture was evacuated and backfilled with hydrogen and then stirred for 18 h. The catalyst was removed via filtration and the solvent was removed *in vacuo* to obtain the crude material which was purified via preparative HPLC to obtain **GEN626** (0.0614 mg, 42% yield) as white solid.

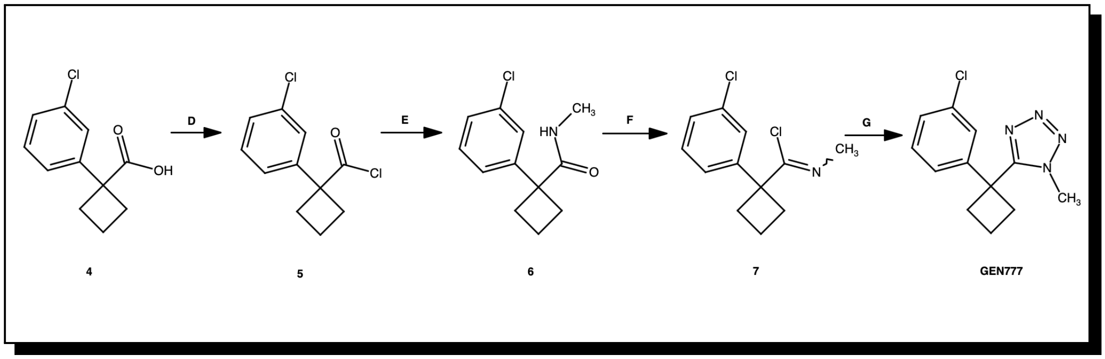

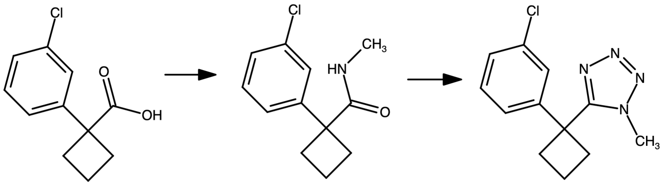

Fig. S4. **GEN777 synthesis route (top) and RXN-predicted retrosynthetic pathway (bottom). (D)** Thionyl chloride (3 g, 25.2 mmol) was added to a solution of compound **4** (1.7 g, 6.6 mmol) in dichloromethane (10 mL) and the mixture was refluxed for 1 h and evaporated under reduced pressure to give compound **5**. **(E)** To a saturated solution of aqueous methylamine (5 g), cooled to 0 °C, was added compound **5** (1.8 g, 7.9 mmol). After the completion of the reaction was confirmed, the resulting mixture was extracted with MTBE. The combined organic layers were washed with brine dried over anhydrous Na_2SO_4 and evaporated under reduced pressure to obtain 1 g of compound **6**, which was used in the next step without further purification. **(F)** To a solution of compound **6** (1 g, 4.5 mmol) in dichloromethane (700 mL) was added PCl_5 (1.4 g, 6.72 mmol). The reaction mixture was stirred for 2 h at r.t. to obtain the solution contained compound **7** which was not isolated but directly used in the next step. **(G)** To the solution of compound **7** in dichloromethane (from **Step F**) was added TMSN3 (2.5 g, 21.7 mmol). The reaction mixture was stirred overnight at r.t. and evaporated under reduced pressure. The residue was purified by HPLC to give 0.130 g of **GEN777**.

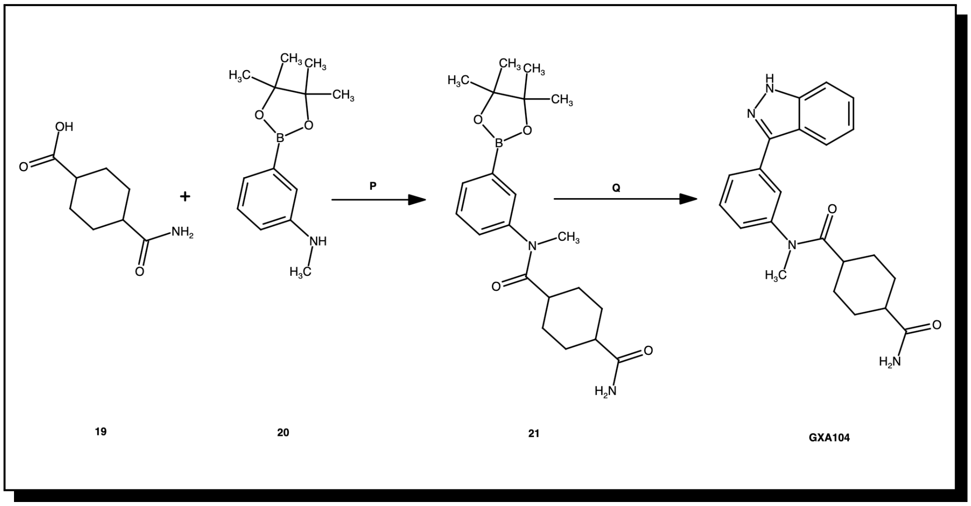

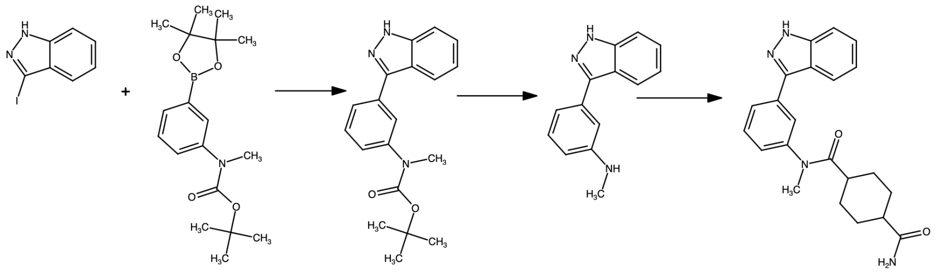

Fig. S5. **GXA104 synthesis route (top) and RXN-predicted retrosynthetic pathway (bottom). (P)** To a solution of compound **19** (0.975 g, 5.70 mmol), compound **20** (1.21 g, 5.18 mmol) and HOBt (0.775 g, 5.70 mmol) in dry DMA (10 mL), cooled to 0 °C, was added dropwise EDC (0.964 g, 6.31 mmol) and the reaction mixture was stirred overnight at r.t., diluted with water and extracted with ethyl acetate. The combined organic layers were washed with water, dried over anhydrous Na_2SO_4 and evaporated under reduced pressure. The residue was crystallized from the minimum amount of ethyl acetate to obtain 1.26 g of compound **21** (63% yield). **(Q)** A solution of compound **21** (0.410 g, 1.06 mmol), 3-iodo-1H-indazole (0.259 g, 1.06 mmol), $Pd(PPh_3)_4$ (0.061 g, 0.05 mmol) and Na_2CO_3 (0.225 g, 2.13 mmol) in a mixture of dioxane/water (4:1) (5 mL) was stirred overnight at 90 °C under an argon atmosphere. The cooled mixture was diluted with water and extracted with dichloromethane. The combined organic layers were washed with water, dried over anhydrous Na_2SO_4 and evaporated under reduced pressure. The residue was purified by column chromatography to obtained by HPLC to afford 0.180 g of compound **GXA104** (45% yield).

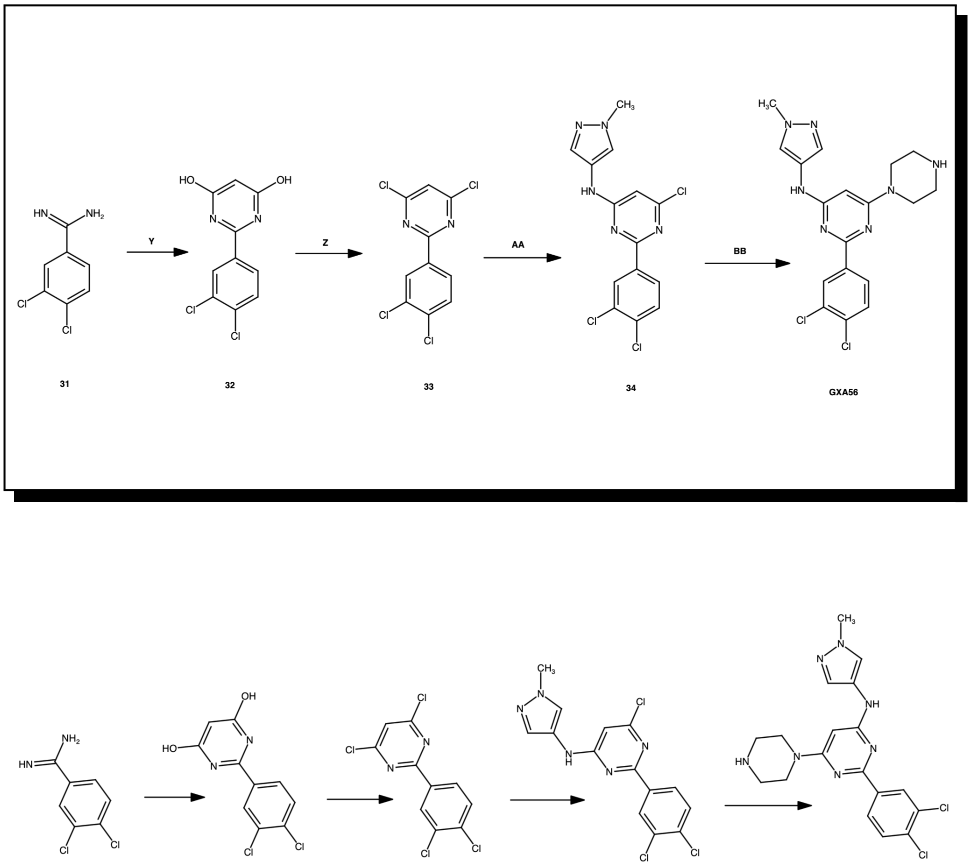

Fig. S6. GXA56 synthesis route (top) and RXN-predicted retrosynthetic pathway (bottom). (Y) Metallic sodium (0.47g, 2.2 eq) was dissolved portionwise in 50 mL of dry methanol. Then **31** (2g, 1eq) and diethylmalonate (1.43g, 1eq) were added thereto. Resulting mixture was stirred at 60 °C overnight. Formed precipitate was filtered off, dissolved in water and acidified with sodium hydrosulphate to pH 2, then stirred for 20 min and filtered to obtain compound **32** as yellow solid. Yield 66%, 1.8 g. **(Z)** To compound **32** (1.8g, 1 eq) in 15 mL of $POCl_3$ was added 0.15 mL of DIPEA and resulting mixture was stirred at reflux for 3 hours. The resulting mixture was evaporated, quenched with ice and saturated solution of anhydrous potassium carbonate up to pH 12. Then the solution was left to stir at ambient temperature for 20 min. The resulting precipitate was filtered off and washed with water several times to obtain compound **33**. Yield 26%, 0.53 g. **(AA)** 1-Methyl-1H-pyrazol-3-amine (0.175 g, 1 eq), sodium iodide (0.27 g, 1 eq) and DIPEA (0.46 g, 2 eq) were added subsequently to a solution of compound **33** (0.5 g, 1 eq) in 10 mL of dry DMF. The resulting mixture was stirred at 80 °C overnight. After mixture was cooled to r.t. and then diluted with water, formed precipitate was filtered and washed with water to give compound **34**. Yield 58%, 0.35g. **(BB)** Compound **34** (0.35 g, 1 eq) together with piperazine (0.17 g, 2 eq) and anhydrous potassium carbonate (0.27 g, 2 eq) was mixed in 15 mL of dry DMF and heated up to 120 °C overnight. Thereafter a mixture was cooled, and insoluble material was filtered out. Then organic layer was evaporated and purified by HPLC to give **GXA56** as a white solid. Yield 22.5%, 0.08g.

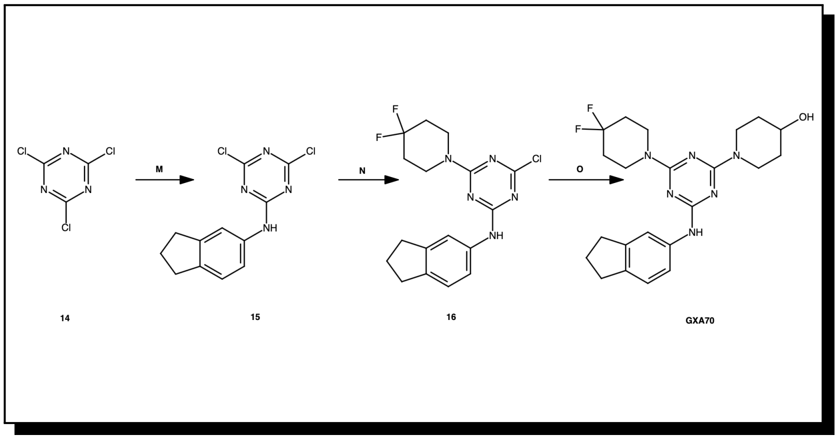

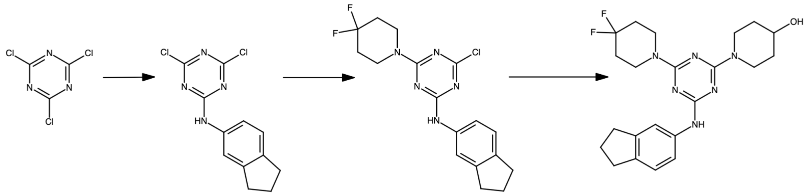

Fig. S7. GXA70 synthesis route (top) and RXN-predicted retrosynthetic pathway (bottom). **(M)** To the solution of compound **14** (2.0 g, 10.8 mmol, 1 eq) in 30 mL of dichloromethane cooled to 0 °C, 1.2 equivalent of DIPEA was added dropwise under continuous stirring. Thereafter 1 eq of 2,3-dihydro-1H-inden-5-amine dissolved in 10 mL of dichloromethane was added. The resulting mixture was stirred at ambient temperature overnight. Thereafter resulting solution was washed with water, 3 × 20 mL. Then organic layer was dried over anhydrous sodium sulfite and evaporated *in vacuo*. Resulting compound **15** with 90% purity was used in the next step without additional purification. Yield 92%, 2.8 g. **(N)** To the solution of compound **15** (2.8 g, 9.7 mmol, 1 eq) in 40 mL of dichloromethane 2.2 equivalents of DIPEA was added dropwise at 0 °C under continuous stirring. The resulting solution was stirred for additional 30 min and then 4,4-difluoropiperidine hydrochloride was added portionwise (1.1 eq). The resulting mixture was left to stir at ambient temperature overnight. Next day the reaction solution was washed with water, 3 × 20 mL. Resulting organic layer was dried with anhydrous sodium disulfite and evaporated under reduced pressure. The resulting product **16** with 90%+ purity was used in the next step without any additional purification. Yield 91%, 3.3 g. **(O)** To the solution of compound **16** (3.3 g, 9.1 mmol, 1 eq) in 40 mL of DMF cooled to 0 °C. 1.2 eq of DIPEA was added dropwise under stirring. Then mixture was stirred for additional 30 min and 1.05 eq of the corresponding amine in 10 mL of DMF was added. Resulting reaction mixture was stirred at 80 °C overnight. Thereafter all volatiles were evaporated *in vacuo* and residue was washed with water twice. Resulting precipitate was dissolved in 50 mL of dichloromethane, dried with anhydrous sodium sulfate and filtered through the Celite pad. Resulting filtrate was evaporated under reduced pressure to give **GXA70** with 95% purity. Yield 70%, 2.7 g.

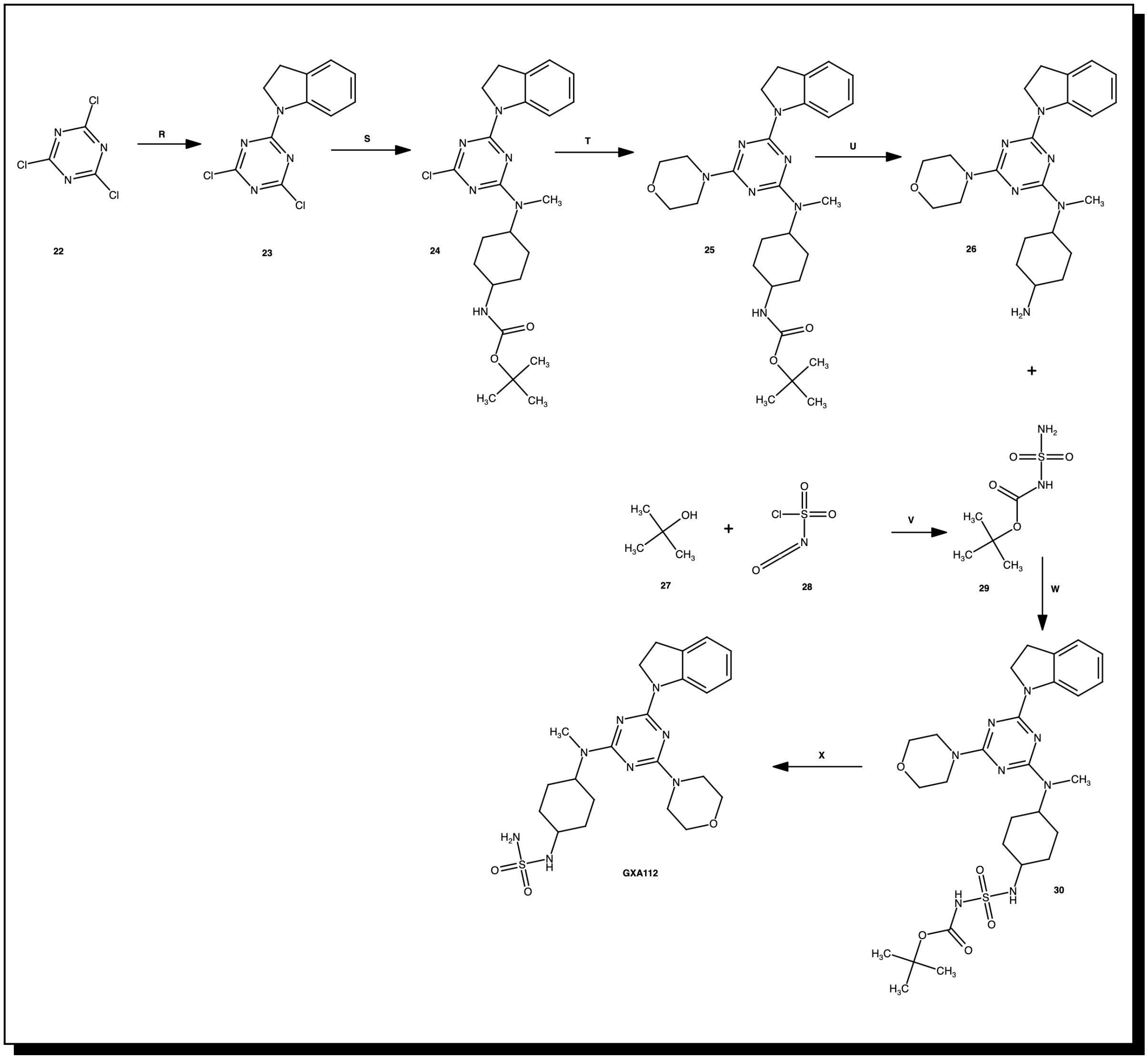

Fig. S8. GXA112 synthesis route. (R) To a stirred solution of compound **22** (2 g, 11 mmol) in dichloromethane (40 mL) at 0 °C were added DIPEA (2.3 mL, 13.2 mmol) and 2,3-dihydro-1H-indole (1.22 mL) and the resulting mixture was stirred at r.t. for 16 h. After that the reaction mixture was diluted with water; the organic phase was washed with water and brine, dried over Na_2SO_4 and evaporated to obtain crude product **23** (1.1 g), which was used in the next step without further purification. **(S)** To a stirred solution of compound **23** (1.1 g, 4 mmol) in dichloromethane (40 mL) at 0 °C were added DIPEA (0.86 mL, 4.94 mmol) and tert-butyl N-[4-(methylamino)cyclohexyl]carbamate (0.94 g) and the resulting mixture was stirred at r.t. for 16 h. After that the reaction mixture was diluted with water; the organic phase was washed with water and brine, dried over Na_2SO_4 and evaporated under reduced pressure to obtain crude product **24** (1.5 g), which was used in the next step without further purification. **(T)** To a stirred solution of compound **24** (1.5 g, 3 mmol) in dichloromethane (30 mL) at r.t. were added DIPEA (0.68 mL, 3.90 mmol) and morpholine (0.28 mL, 3.25 mmol) and the resulting mixture was stirred at r.t. for 16 h. After that an additional amount of DIPEA (0.68 mL, 3.90 mmol) and morpholine (0.28 mL, 3.25 mmol) was added and the resulting mixture was stirred at r.t. for another 16 h. Then the reaction mixture was diluted with water; the organic phase was washed with water and brine, dried over Na_2SO_4 and evaporated under reduced pressure to obtain crude product **25** (1.7 g), which was used in the next step without further purification. **(U)** To a stirred solution of compound **25** (1.7 g, 3 mmol) in dichloromethane (25 mL) was added 4 M HCl solution in dioxane and the resulting mixture was stirred at r.t. for 8 h. After that the reaction mixture was evaporated under reduced pressure to obtain crude product **26** (1.2 g), which was used in the next step without further purification. **(V)** To a stirred solution of compound **27** (0.7 mL, 7.4 mmol) in diethyl ether (10 mL) was added compound **28** (0.15 mL, 0.243 g, 1.7 mmol) at −78 °C and the resulting mixture was stirred at r.t. for 1 h. The reaction mixture was evaporated without heating to obtain crude product **29**, which was immediately used in the next step.

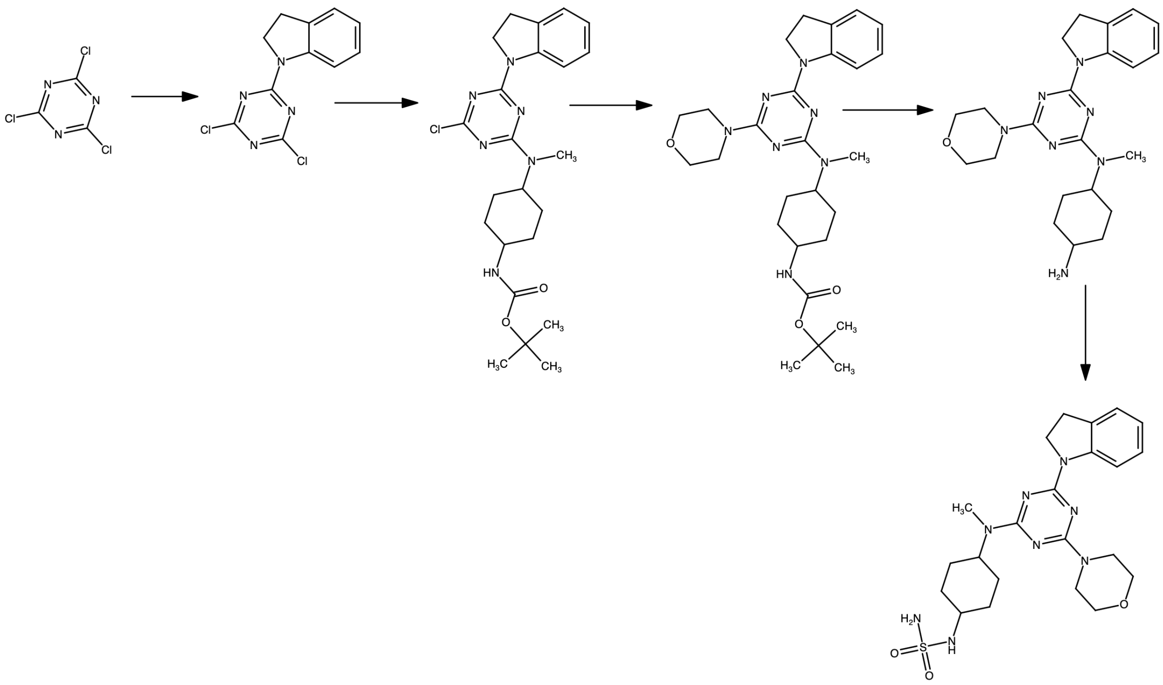

Fig. S8 (continued). GXA112 RXN-predicted retrosynthetic pathway. (W) To a stirred suspension of compound **26** (0.8 g, 1.7 mmol) in dichloromethane (10 mL) at 0 °C was added Et_3N (0.76 mL, 5.45 mmol) followed by a solution of compound **29** in dichloromethane (3 mL) and the resulting mixture was stirred at r.t. for 16 h. After that the reaction mixture was diluted with water; the organic phase was washed with water and brine, dried over Na_2SO_4 and evaporated under reduced pressure to obtain crude product **30** (0.8 g), which was used in the next step without further purification. **(X)** To a stirred solution of compound **30** (0.8 g, 1.4 mmol) in dichloromethane (5 mL) was added 4 M HCl solution in dioxane (1 mL) and the resulting mixture was stirred at r.t. for 8 h. Then the reaction mixture was evaporated under reduced pressure, the obtained residue was diluted with water, basified with a $NaHCO_3$ solution and extracted with dichloromethane. The combined organic phase was washed with water, dried over Na_2SO_4 and evaporated under reduced pressure to obtain crude product. The crude product was purified by HPLC to obtain 0.01 g of **GXA112**.

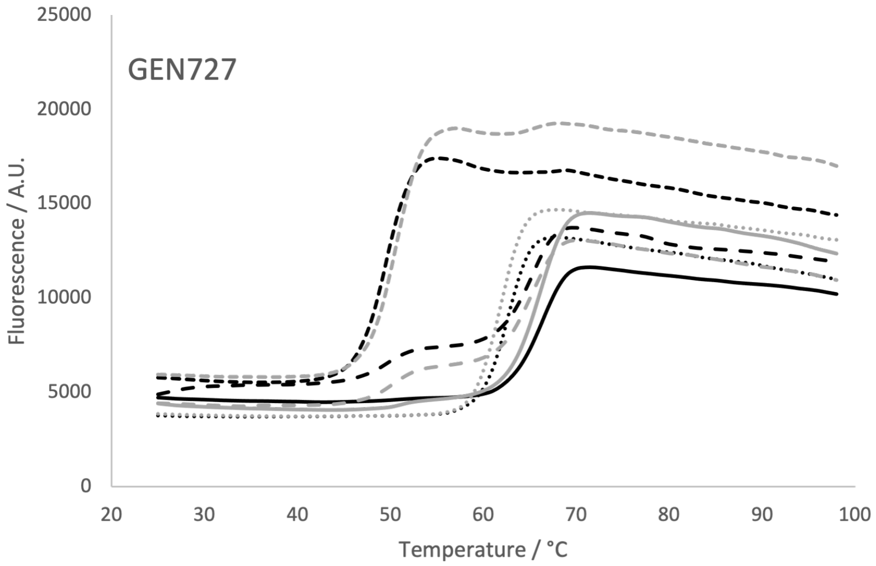

Fig. S9. Thermofluor assay results. Thermofluor raw fluorescence data for experiments with AI-designed compound GEN727 (black) and a DMSO control (grey). Data were recorded using protein that was used immediately after dilution into neutral buffer (solid lines), incubated overnight in neutral buffer (long-dashed lines), or incubated overnight with the compound in neutral buffer (short-dashed lines). For comparison, data from protein in pH 4.6 buffer is also shown (dotted lines).

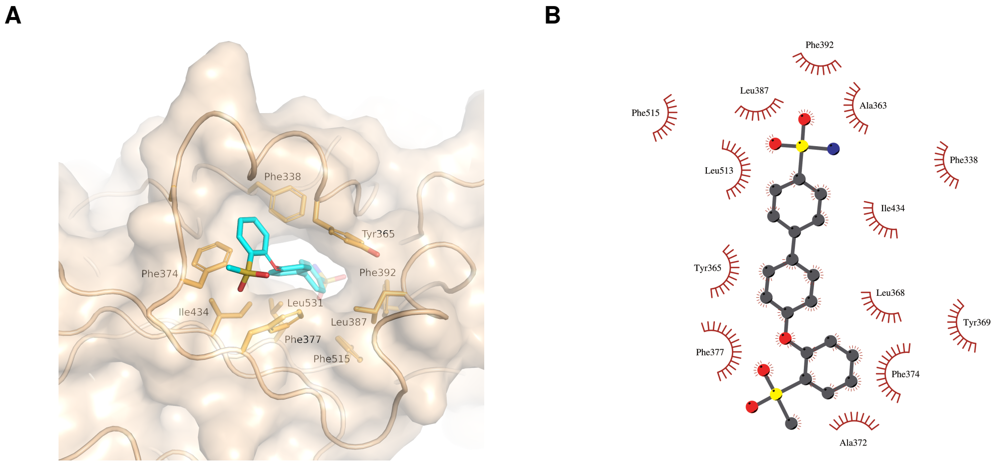

Fig. S10. Docked structure of SARS-CoV-2 spike protein RBD in complex with GEN725. (A) Surface representation depicting the overall ligand binding modes of GEN725 at the lipid binding site of the RBD. **(B)** Schematic representation of the ligand interactions with the spike RBD.

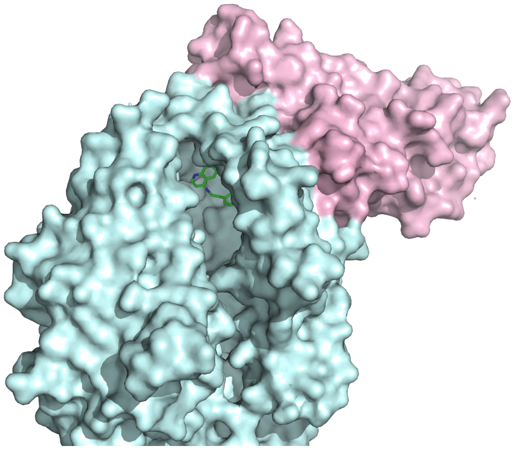

Fig. S11. Docked structure of human ACE2 (cyan) in complex with GEN727 (green). SARS-CoV-2 spike RBD is also shown in pink.

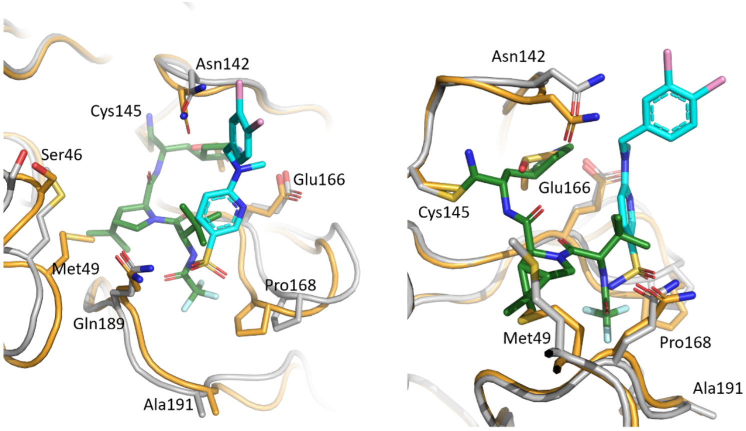

Fig. S12. Comparison of crystal structures of Z68337194 and nirmatrelvir. SARS-CoV-2 M^{pro} in complex with Z68337194 (protein chain in orange, ligand in cyan), aligned to SARS-CoV-2 M^{pro} in complex with nirmatrelvir (7TE0, protein in gray, ligand in green). Images are related by a 90° rotation around the z-axis.

Molecule 1

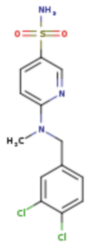

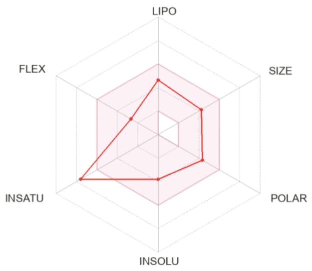

SMILES CN(c1ccc(cn1)S(=O)(=O)N)Cc1ccc(c(c1)Cl)Cl

Physicochemical Properties	
Formula	C13H13Cl2N3O2S
Molecular weight	346.23 g/mol
Num. heavy atoms	21
Num. arom. heavy atoms	12
Fraction Csp3	0.15
Num. rotatable bonds	4
Num. H-bond acceptors	4
Num. H-bond donors	1
Molar Refractivity	83.95
TPSA	84.67 Å²

Lipophilicity	
Log $P_{o/w}$ (iLOGP)	2.29
Log $P_{o/w}$ (XLOGP3)	2.62
Log $P_{o/w}$ (WLOGP)	3.60
Log $P_{o/w}$ (MLOGP)	1.85
Log $P_{o/w}$ (SILICOS-IT)	1.82
Consensus Log $P_{o/w}$	2.44

Water Solubility	
Log S (ESOL)	-3.80
Solubility	5.54e-02 mg/ml ; 1.60e-04 mol/l
Class	Soluble
Log S (Ali)	-4.05
Solubility	3.10e-02 mg/ml ; 8.95e-05 mol/l
Class	Moderately soluble
Log S (SILICOS-IT)	-5.43
Solubility	1.28e-03 mg/ml ; 3.70e-06 mol/l
Class	Moderately soluble

Pharmacokinetics	
GI absorption	High
BBB permeant	No
P-gp substrate	No
CYP1A2 inhibitor	Yes
CYP2C19 inhibitor	Yes
CYP2C9 inhibitor	Yes
CYP2D6 inhibitor	No
CYP3A4 inhibitor	No
Log K_p (skin permeation)	-6.55 cm/s

Druglikeness	
Lipinski	Yes; 0 violation
Ghose	Yes
Veber	Yes
Egan	Yes
Muegge	Yes
Bioavailability Score	0.55

Medicinal Chemistry	
PAINS	0 alert
Brenk	0 alert
Leadlikeness	Yes
Synthetic accessibility	2.44

Fig. S13. SwissADME evaluation of Z68337194.

Molecule 4

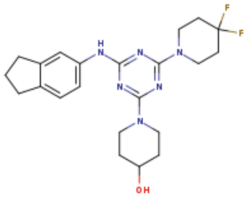

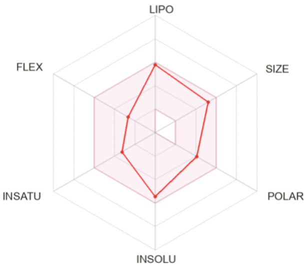

SMILES OC1CCN(CC1)c1nc(Nc2ccc3c(c2)CCC3)nc(n1)N1CCC(CC1)(F)F

Physicochemical Properties	
Formula	C22H28F2N6O
Molecular weight	430.49 g/mol
Num. heavy atoms	31
Num. arom. heavy atoms	12
Fraction Csp3	0.59
Num. rotatable bonds	4
Num. H-bond acceptors	6
Num. H-bond donors	2
Molar Refractivity	122.20
TPSA	77.41 Å²

Lipophilicity	
Log $P_{o/w}$ (iLOGP)	3.70
Log $P_{o/w}$ (XLOGP3)	4.63
Log $P_{o/w}$ (WLOGP)	3.38
Log $P_{o/w}$ (MLOGP)	3.05
Log $P_{o/w}$ (SILICOS-IT)	2.70
Consensus Log $P_{o/w}$	3.49

Water Solubility	
Log *S* (ESOL)	-5.45
Solubility	1.53e-03 mg/ml ; 3.56e-06 mol/l
Class	Moderately soluble
Log *S* (Ali)	-5.98
Solubility	4.49e-04 mg/ml ; 1.04e-06 mol/l
Class	Moderately soluble
Log *S* (SILICOS-IT)	-5.76
Solubility	7.41e-04 mg/ml ; 1.72e-06 mol/l
Class	Moderately soluble

Pharmacokinetics	
GI absorption	High
BBB permeant	Yes
P-gp substrate	No
CYP1A2 inhibitor	No
CYP2C19 inhibitor	Yes
CYP2C9 inhibitor	Yes
CYP2D6 inhibitor	Yes
CYP3A4 inhibitor	No
Log K_p (skin permeation)	-5.64 cm/s

Druglikeness	
Lipinski	Yes; 0 violation
Ghose	Yes
Veber	Yes
Egan	Yes
Muegge	Yes
Bioavailability Score	0.55

Medicinal Chemistry	
PAINS	0 alert
Brenk	0 alert
Leadlikeness	No; 2 violations: MW>350, XLOGP3>3.5
Synthetic accessibility	3.22

Fig. S14. **SwissADME evaluation of GXA70.**

Molecule 6

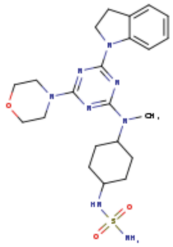

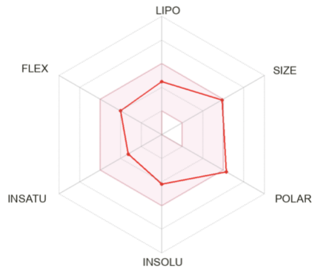

SMILES CN(c1nc(nc(n1)N1CCc2c1cccc2)N1CCOCC1)C1CCC(CC1)NS(=O)(=O)N

Physicochemical Properties	
Formula	C22H32N8O3S
Molecular weight	488.61 g/mol
Num. heavy atoms	34
Num. arom. heavy atoms	12
Fraction Csp3	0.59
Num. rotatable bonds	6
Num. H-bond acceptors	8
Num. H-bond donors	2
Molar Refractivity	136.39
TPSA	138.19 Å²

Lipophilicity	
Log $P_{o/w}$ (iLOGP)	2.68
Log $P_{o/w}$ (XLOGP3)	2.33
Log $P_{o/w}$ (WLOGP)	1.26
Log $P_{o/w}$ (MLOGP)	1.61
Log $P_{o/w}$ (SILICOS-IT)	-1.00
Consensus Log $P_{o/w}$	1.38

Water Solubility	
Log *S* (ESOL)	-4.20
Solubility	3.07e-02 mg/ml ; 6.27e-05 mol/l
Class	Moderately soluble
Log *S* (Ali)	-4.87
Solubility	6.58e-03 mg/ml ; 1.35e-05 mol/l
Class	Moderately soluble
Log *S* (SILICOS-IT)	-4.25
Solubility	2.73e-02 mg/ml ; 5.58e-05 mol/l
Class	Moderately soluble

Pharmacokinetics	
GI absorption	High
BBB permeant	No
P-gp substrate	Yes
CYP1A2 inhibitor	No
CYP2C19 inhibitor	Yes
CYP2C9 inhibitor	No
CYP2D6 inhibitor	Yes
CYP3A4 inhibitor	Yes
Log K_p (skin permeation)	-7.63 cm/s

Druglikeness	
Lipinski	Yes; 1 violation: NorO>10
Ghose	No; 2 violations: MW>480, MR>130
Veber	Yes
Egan	No; 1 violation: TPSA>131.6
Muegge	Yes
Bioavailability Score	0.55

Medicinal Chemistry	
PAINS	0 alert
Brenk	0 alert
Leadlikeness	No; 1 violation: MW>350
Synthetic accessibility	4.34

Fig. S15. SwissADME evaluation of GXA112.

Molecule 7

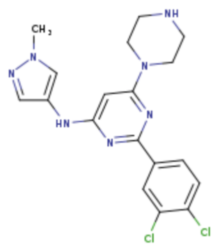

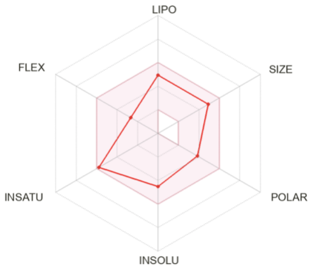

SMILES Cn1ncc(c1)Nc1cc(nc(n1)c1ccc(c(c1)Cl)Cl)N1CCNCC1

Physicochemical Properties	
Formula	C18H19Cl2N7
Molecular weight	404.30 g/mol
Num. heavy atoms	27
Num. arom. heavy atoms	17
Fraction Csp3	0.28
Num. rotatable bonds	4
Num. H-bond acceptors	4
Num. H-bond donors	2
Molar Refractivity	115.44
TPSA	70.90 Å²

Lipophilicity	
Log $P_{o/w}$ (iLOGP)	3.20
Log $P_{o/w}$ (XLOGP3)	3.11
Log $P_{o/w}$ (WLOGP)	2.58
Log $P_{o/w}$ (MLOGP)	2.74
Log $P_{o/w}$ (SILICOS-IT)	2.44
Consensus Log $P_{o/w}$	2.81

Water Solubility	
Log *S* (ESOL)	-4.51
Solubility	1.26e-02 mg/ml ; 3.11e-05 mol/l
Class	Moderately soluble
Log *S* (Ali)	-4.27
Solubility	2.18e-02 mg/ml ; 5.40e-05 mol/l
Class	Moderately soluble
Log *S* (SILICOS-IT)	-6.79
Solubility	6.62e-05 mg/ml ; 1.64e-07 mol/l
Class	Poorly soluble

Pharmacokinetics	
GI absorption	High
BBB permeant	Yes
P-gp substrate	Yes
CYP1A2 inhibitor	Yes
CYP2C19 inhibitor	No
CYP2C9 inhibitor	Yes
CYP2D6 inhibitor	Yes
CYP3A4 inhibitor	Yes
Log K_p (skin permeation)	-6.56 cm/s

Druglikeness	
Lipinski	Yes; 0 violation
Ghose	Yes
Veber	Yes
Egan	Yes
Muegge	Yes
Bioavailability Score	0.55

Medicinal Chemistry	
PAINS	0 alert
Brenk	0 alert
Leadlikeness	No; 1 violation: MW>350
Synthetic accessibility	3.11

Fig. S16. **SwissADME evaluation of GXA56.**

Molecule 9

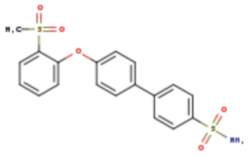

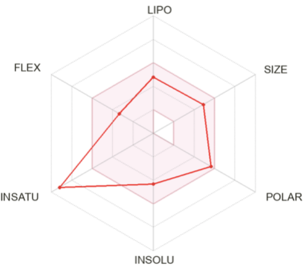

SMILES NS(=O)(=O)c1ccc(cc1)c1ccc(cc1)Oc1ccccc1S(=O)(=O)C

Physicochemical Properties	
Formula	C19H17NO5S2
Molecular weight	403.47 g/mol
Num. heavy atoms	27
Num. arom. heavy atoms	18
Fraction Csp3	0.05
Num. rotatable bonds	5
Num. H-bond acceptors	6
Num. H-bond donors	1
Molar Refractivity	102.48
TPSA	120.29 Å²

Lipophilicity	
Log $P_{o/w}$ (iLOGP)	1.79
Log $P_{o/w}$ (XLOGP3)	2.88
Log $P_{o/w}$ (WLOGP)	5.36
Log $P_{o/w}$ (MLOGP)	2.43
Log $P_{o/w}$ (SILICOS-IT)	2.02
Consensus Log $P_{o/w}$	2.90

Water Solubility	
Log *S* (ESOL)	-4.32
Solubility	1.93e-02 mg/ml ; 4.79e-05 mol/l
Class	Moderately soluble
Log *S* (Ali)	-5.07
Solubility	3.47e-03 mg/ml ; 8.59e-06 mol/l
Class	Moderately soluble
Log *S* (SILICOS-IT)	-6.97
Solubility	4.33e-05 mg/ml ; 1.07e-07 mol/l
Class	Poorly soluble

Pharmacokinetics	
GI absorption	Low
BBB permeant	No
P-gp substrate	No
CYP1A2 inhibitor	No
CYP2C19 inhibitor	Yes
CYP2C9 inhibitor	Yes
CYP2D6 inhibitor	No
CYP3A4 inhibitor	No
Log K_p (skin permeation)	-6.72 cm/s

Druglikeness	
Lipinski	Yes; 0 violation
Ghose	Yes
Veber	Yes
Egan	Yes
Muegge	Yes
Bioavailability Score	0.55

Medicinal Chemistry	
PAINS	0 alert
Brenk	0 alert
Leadlikeness	No; 1 violation: MW>350
Synthetic accessibility	3.10

Fig. S17. SwissADME evaluation of GEN725.

Molecule 10

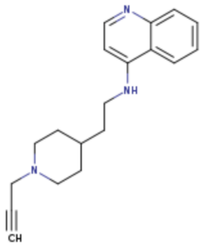

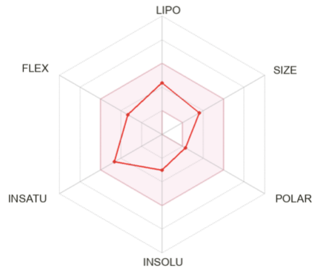

SMILES C#CCN1CCC(CC1)CCNc1ccnc2c1cccc2

Physicochemical Properties	
Formula	C19H23N3
Molecular weight	293.41 g/mol
Num. heavy atoms	22
Num. arom. heavy atoms	10
Fraction Csp3	0.42
Num. rotatable bonds	5
Num. H-bond acceptors	2
Num. H-bond donors	1
Molar Refractivity	97.17
TPSA	28.16 Å²

Lipophilicity	
Log $P_{o/w}$ (iLOGP)	3.40
Log $P_{o/w}$ (XLOGP3)	2.14
Log $P_{o/w}$ (WLOGP)	2.89
Log $P_{o/w}$ (MLOGP)	2.86
Log $P_{o/w}$ (SILICOS-IT)	3.59
Consensus Log $P_{o/w}$	2.98

Water Solubility	
Log *S* (ESOL)	-3.01
Solubility	2.84e-01 mg/ml ; 9.69e-04 mol/l
Class	Soluble
Log *S* (Ali)	-2.36
Solubility	1.27e+00 mg/ml ; 4.33e-03 mol/l
Class	Soluble
Log *S* (SILICOS-IT)	-5.54
Solubility	8.56e-04 mg/ml ; 2.92e-06 mol/l
Class	Moderately soluble

Pharmacokinetics	
GI absorption	High
BBB permeant	Yes
P-gp substrate	Yes
CYP1A2 inhibitor	Yes
CYP2C19 inhibitor	No
CYP2C9 inhibitor	No
CYP2D6 inhibitor	Yes
CYP3A4 inhibitor	No
Log K_p (skin permeation)	-6.57 cm/s

Druglikeness	
Lipinski	Yes; 0 violation
Ghose	Yes
Veber	Yes
Egan	Yes
Muegge	Yes
Bioavailability Score	0.55

Medicinal Chemistry	
PAINS	0 alert
Brenk	1 alert: triple_bond
Leadlikeness	Yes
Synthetic accessibility	2.29

Fig. S18. **SwissADME evaluation of GEN727.**

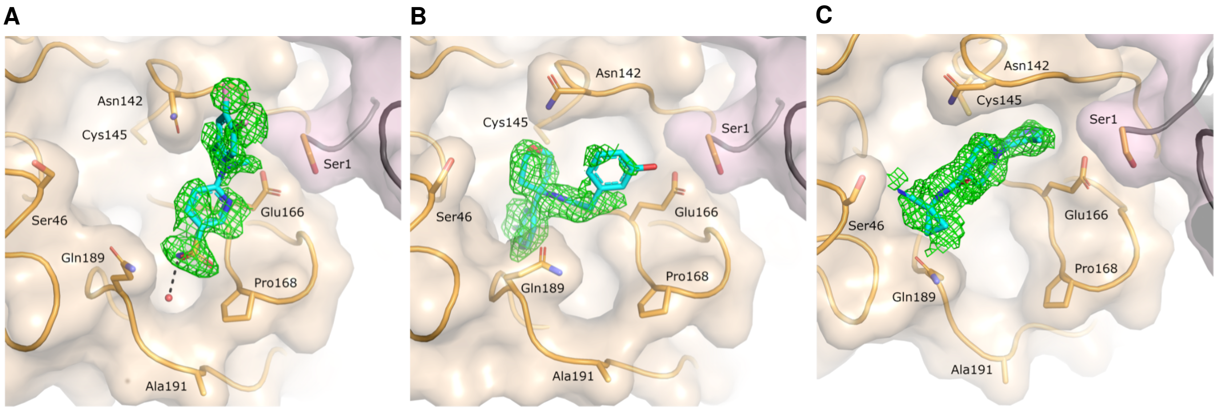

Fig. S19. Pan Dataset Density Analysis (PanDDA) event maps. PanDDA[78] event maps for crystal structures of the SARS-CoV-2 M^{pro} in complex with **(A)** Z6833714, **(B)** Z1633315555, and **(C)** Z1365651030. All event maps are contoured at the 1σ level. The PanDDA algorithm facilitates identification of weakly bound ligands as described previously[73].

Supplementary Text

Algorithm S1 Conditional Latent (attribute) Space Sampling (CLaSS)

Require: Trained latent variable model (e.g. VAE), samples $\mathbf{z}_j$ drawn from domain of interest, labeled samples for each attribute a_i.

Encode training data $\mathbf{x}_j$ in latent space: $\mathbf{z}_{j,k} \sim q_\phi(\mathbf{z}|\mathbf{x}_j)$ for $k = 1, ..., K$

Use $\mathbf{z}_{j,k}$ to fit explicit density model $Q_\xi(\mathbf{z})$ to approximate marginal posterior $q_\phi(\mathbf{z})$

Train classifier models $q_\xi(a_i|\mathbf{z})$ using labeled samples for each attribute a_i to approximate probability $p(a_i|\mathbf{x})$

Assuming attributes a_i are conditionally independent given $\mathbf{z}$, then

$$\hat{p_\xi}(\mathbf{z}|\mathbf{a}) = \frac{Q_\xi(\mathbf{z}) \prod_i q_\xi(a_i|\mathbf{z})}{q_\xi(\mathbf{z})}$$

via Bayes' rule.

Let $g(\mathbf{z}) = Q_\xi(\mathbf{z})$ and $M = \frac{1}{q_\xi(\mathbf{a})}$

repeat

    Sample from $Q_\xi(\mathbf{z})$

    Accept with probability $\frac{f(\mathbf{z})}{Mg(\mathbf{z})} = \prod_i q_\xi(a_i|\mathbf{z}) \leq 1$

    if Accepted **then**

        Decode sample from latent and save: $\mathbf{x} \sim p_\theta(\mathbf{x}|\mathbf{z})$

    end if

until Desired number of samples attained

return Accepted samples

Additional supplementary files associated with this manuscript include:

5SML PanDDA event files (.zip)

5SMM PanDDA event files (.zip)

5SMN PanDDA event files (.zip)

Supplementary code (.zip)

COVID-19 Molecule Explorer (Mpro) (.csv)

COVID-19 Molecule Explorer (RBD) (.csv)

REFERENCES AND NOTES

1. M. D. Lloyd, High-throughput screening for the discovery of enzyme inhibitors. *J. Med. Chem.* **63**, 10742–10772 (2020).
2. P. G. Polishchuk, T. I. Madzhidov, A. Varnek, Estimation of the size of drug-like chemical space based on GDB-17 data. *J. Comput. Aided Mol. Des.* **27**, 675–679 (2013).
3. J. A. DiMasi, H. G. Grabowski, R. W. Hansen, Innovation in the pharmaceutical industry: New estimates of R&D costs. *J. Health Econ.* **47**, 20–33 (2016).
4. A. Zunger, Inverse design in search of materials with target functionalities. *Nat. Rev. Chem.* **2**, 1–16 (2018).
5. T. Sousa, J. Correia, V. Pereira, M. Rocha, Generative deep learning for targeted compound design. *J. Chem. Inf. Model.* **61**, 5343–5361 (2021).
6. A. Zhavoronkov, Y. A. Ivanenkov, A. Aliper, M. S. Veselov, V. A. Aladinskiy, A. V. Aladinskaya, V. A. Terentiev, D. A. Polykovskiy, M. D. Kuznetsov, A. Asadulaev, Y. Volkov, A. Zholus, R. R. Shayakhmetov, A. Zhebrak, L. I. Minaeva, B. A. Zagribelnyy, L. H. Lee, R. Soll, D. Madge, L. Xing, T. Guo, A. Aspuru-Guzik, Deep learning enables rapid identification of potent DDR1 kinase inhibitors. *Nat. Biotechnol.* **37**, 1038–1040 (2019).
7. D. Merk, L. Friedrich, F. Grisoni, G. Schneider, De novo design of bioactive small molecules by artificial intelligence. *Mol. informatics* **37**, 1700153 (2018).
8. F. Grisoni, B. J. H. Huisman, A. L. Button, M. Moret, K. Atz, D. Merk, G. Schneider, Combining generative artificial intelligence and on-chip synthesis for de novo drug design. *Sci. Adv.* **7**, eabg3338 (2021).
9. V. Chenthamarakshan, P. Das, S. C. Hoffman, H. Strobelt, I. Padhi, K. W. Lim, B. Hoover, M. Manica, J. Born, T. Laino, A. Mojsilovic, CogMol: Target-specific and selective drug design for COVID-19 using deep generative models. arXiv:2004.01215 [cs.LG] (2 April 2020).
10. R. Bommasani, D. A. Hudson, E. Adeli, R. Altman, S. Arora, Sydney von Arx, M. S. Bernstein, J. Bohg, A. Bosselut, E. Brunskill, E. Brynjolfsson, S. Buch, D. Card, R. Castellon, N. Chatterji, A. Chen, K. Creel, J. Q. Davis, D. Demszky, C. Donahue, M. Doumbouya, E. Durmus, S. Ermon, J. Etchemendy, K. Ethayarajh, L. Fei-Fei, C. Finn, T. Gale, L. Gillespie, K. Goel, N. Goodman, S. Grossman, N. Guha, T. Hashimoto, P. Henderson, J. Hewitt, D. E. Ho, J. Hong, K. Hsu, J. Huang, T. Icard, S. Jain, D. Jurafsky, P. Kalluri, S. Karamcheti, G. Keeling, F. Khani, O. Khattab, P. W. Koh, M. Krass, R. Krishna, R. Kuditipudi, A. Kumar, F. Ladhak, M. Lee, T. Lee, J. Leskovec, I. Levent, X. L. Li, X. Li, T. Ma, A.

Malik, C. D. Manning, S. Mirchandani, E. Mitchell, Z. Munyikwa, S. Nair, A. Narayan, D. Narayanan, B. Newman, A. Nie, J. C. Niebles, H. Nilforoshan, J. Nyarko, G. Ogut, L. Orr, I. Papadimitriou, J. S. Park, C. Piech, E. Portelance, C. Potts, A. Raghunathan, R. Reich, H. Ren, F. Rong, Y. Roohani, C. Ruiz, J. Ryan, C. Ré, D. Sadigh, S. Sagawa, K. Santhanam, A. Shih, K. Srinivasan, A. Tamkin, R. Taori, A. W. Thomas, F. Tramèr, R. E. Wang, W. Wang, B. Wu, J. Wu, Y. Wu, S. M. Xie, M. Yasunaga, J. You, M. Zaharia, M. Zhang, T. Zhang, X. Zhang, Y. Zhang, L. Zheng, K. Zhou, P. Liang, On the opportunities and risks of foundation models. arXiv:2108.07258 [cs.LG] (16 August 2021).

11. IBM, What are foundation models? (2022) [accessed May 2022].
12. IBM, CogMol Molecule Explorer; https://covid19-mol.vizhub.ai/ [released April 2020; accessed 7 March 2022].
13. D. P. Kingma, M. Welling, Auto-encoding variational Bayes. arXiv:1312.6114 [stat.ML] (20 December 2013).
14. D. Weininger, SMILES, a chemical language and information system. 1. Introduction to methodology and encoding rules. *J. Chem. Inf. Comput. Sci.* **28**, 31–36 (1988).
15. E. C. Alley, G. Khimulya, S. Biswas, M. AlQuraishi, G. M. Church, Unified rational protein engineering with sequence-based deep representation learning. *Nat. Methods* **16**, 1315–1322 (2019).
16. P. Das, T. Sercu, K. Wadhawan, I. Padhi, S. Gehrmann, F. Cipcigan, V. Chenthamarakshan, H. Strobelt, P.-Y. Cicero dos Santos, Y. Y. Chen, J. P. K. Yang, J. Tan J. Hedrick, A. M. Crain, Accelerated antimicrobial discovery via deep generative models and molecular dynamics simulations. *Nat. Biomed. Eng.* **5**, 613–623 (2021).
17. P. Schwaller, R. Petraglia, V. Zullo, V. H. Nair, R. A. Haeuselmann, R. Pisoni, C. Bekas, A. Iuliano, T. Laino, Predicting retrosynthetic pathways using transformer-based models and a hyper-graph exploration strategy. *Chem. Sci.* **11**, 3316–3325 (2020).
18. C. Toelzer, K. Gupta, S. K. N. Yadav, U. Borucu, A. D. Davidson, M. K. Williamson, D. K. Shoemark, F. Garzoni, O. Staufer, R. Milligan, J. Capin, A. J. Mulholland, J. Spatz, D. Fitzgerald, I. Berger, C. Schaffitzel, Free fatty acid binding pocket in the locked structure of sars-cov-2 spike protein. *Science* **370**, 725–730 (2020).
19. L. Carrique, H. M. Duyvesteyn, T. Malinauskas, Y. Zhao, J. Ren, D. Zhou, T. S. Walter, J. Radecke, J. Huo, R. R. Ruza, P. N. Shah, E. E. Fry, D. I. Stuart, The sars-cov-2 spike harbours a lipid binding pocket which modulates stability of the prefusion trimer. bioRxiv 2020.08.13.249177 [**Preprint**] (13 August 2020).

20. S. Oskar, G. Kapil, B. J. E. Hernandez, K. Fabian, S. Christian, S. Gunjita, V. Kate, R. A. Yagüe, M. Meline, F. Sebastian, D. Hendrik, A. E. A. Cavalcanti, S. Christiane, R. Alessia, P. Ilia, I. Berger, J. P. Spatz, Synthetic virions reveal fatty acid-coupled adaptive immunogenicity of sars-cov-2 spike glycoprotein. *Nat. Commun.* **13**, 1–13 (2022).
21. C. Toelzer, K. Gupta, S. K. N. Yadav, L. Hodgson, M. K. Williamson, D. Buzas, U. Borucu, K. Powers, R. Stenner, K. Vasileiou, F. Garzoni, D. Fitzgerald, C. Payré, G. Gautam, G. Lambeau, A. D. Davidson, P. Verkade, M. Frank, I. Berger, C. Schaffitzel, The free fatty acid-binding pocket is a conserved hallmark in pathogenic β-coronavirus spike proteins from sars-cov to omicron. *Sci. Adv.* **8**, eadc9179 (2022).
22. R. Creutznacher, T. Maass, B. Veselkova, G. Ssebyatika, T. Krey, M. Empting, N. Tautz, M. Frank, K. Kölbel, C. Uetrecht, T. Peters, Nmr experiments provide insights into ligand-binding to the sars-cov-2 spike protein receptor-binding domain. *J. Am. Chem. Soc.* **144**, 13060–13065 (2022).
23. C. J. Day, B. Bailly, P. Guillon, L. Dirr, F. E.C. Jen, B. L. Spillings, J. Mak, M. von Itzstein, T. Haselhorst, M. P. Jennings, Multidisciplinary approaches identify compounds that bind to human ace2 or sars-cov-2 spike protein as candidates to block sars-cov-2–Ace2 receptor interactions. *mBio* **12**, e03681–20 (2021).
24. A. Goc, A. Niedzwiecki, M. Rath, Polyunsaturated ω-3 fatty acids inhibit ace2-controlled sars-cov-2 binding and cellular entry. *Sci. Rep.* **11**, 1–12 (2021).
25. T. R. Malla, A. Tumber, T. John, L. Brewitz, C. Strain-Damerell, C David Owen, P. Lukacik, H T Henry Chan, P. Maheswaran, E. Salah, F. Duarte, H. Yang, Z. Rao, M. A. Walsh, C. J. Schofield, Mass spectrometry reveals potential of β-lactams as sars-cov-2 m pro inhibitors. *Chem. Commun.* **57**, 1430–1433 (2021).
26. A. Morris, W. M. Corkindale; The COVID Moonshot Consortium, N. Drayman, J. D. Chodera, S. Tay, N. London, A. A. Lee, Discovery of sars-cov-2 main protease inhibitors using a synthesis-directed de novo design model. *Chem. Commun.* **57**, 5909–5912 (2021).
27. E. Glaab, G. B. Manoharan, D. Abankwa, Pharmacophore model for sars-cov-2 3CLpro small-molecule inhibitors and in vitro experimental validation of computationally screened inhibitors. *J. Chem. Inf. Model.* **61**, 4082–4096 (2021).
28. C.-H. Zhang, E. A. Stone, M. Deshmukh, J. A. Ippolito, M. M. Ghahremanpour, J. Tirado-Rives, K. A. Spasov, S. Zhang, Y. Takeo, S. N. Kudalkar, Z. Liang, F. Isaacs, B. Lindenbach, S. J. Miller, K. S. Anderson, W. L. Jorgensen, Potent noncovalent inhibitors of the main protease of sars-cov-2 from

molecular sculpting of the drug perampanel guided by free energy perturbation calculations. *ACS Central Sci.* **7**, 467–475 (2021).

29. M. Moret, I. P. Angona, L. Cotos, S. Yan, K. Atz, C. Brunner, M. Baumgartner, F. Grisoni, G. Schneider, Leveraging molecular structure and bioactivity with chemical language models for de novo drug design. *Nat. Commun.* **14**, 1–12 (2023).
30. Enamine, Enamine Advanced Collection; https://enamine.net/compound-collections/screening-collection/ advanced-collection (2022) [accessed 7 March 2022].
31. Z. Jin, X. Du, Y. Xu, Y. Deng, M. Liu, Y. Zhao, B. Zhang, X. Li, L. Zhang, C. Peng, Y. Duan, J. Yu, L. Wang, K. Yang, F. Liu, R. Jiang, X. Yang, T. You, X. Liu, X. Yang, F. Bai, H. Liu, X. Liu, L. W. Guddat, W. Xu, G. Xiao, C. Qin, Z. Shi, H. Jiang, Z. Rao, H. Yang, Structure of mpro from SARS-CoV-2 and discovery of its inhibitors. *Nature* **582**, 289–293 (2020).
32. R. Abdelnabi, C. S. Foo, D. Jochmans, L. Vangeel, S. De Jonghe, P. Augustijns, R. Mols, B. Weynand, T. Wattanakul, R. M. Hoglund, J. Tarning, C. E. Mowbray, P. Sjö, F. Escudié, I. Scandale, E. Chatelain, J. Neyts, The oral protease inhibitor (pf-07321332) protects syrian hamsters against infection with sars-cov-2 variants of concern. *Nat. Commun.* **13**, 719 (2022).
33. S. Durdagi, Ç. Dağ, B. Dogan, M. Yigin, T. Avsar, C. Buyukdag, I. Erol, F. B. Ertem, S. Calis, G. Yildirim, M. D. Orhan, O. Guven, B. Aksoydan, E. Destan, K. Sahin, S. O. Besler, L. Oktay, A. Shafiei, I. Tolu, E. Ayan, B. Yuksel, A. B. Peksen, O. Gocenler, A. D. Yucel, O. Can, S. Ozabrahamyan, A. Olkan, E. Erdemoglu, F. Aksit, G. Tanisali, O. M. Yefanov, A. Barty, A. Tolstikova, G. K. Ketawala, S. Botha, E Han Dao, B. Hayes, M. Liang, M. H. Seaberg, M. S. Hunter, A. Batyuk, V. Mariani, Z. Su, F. Poitevin, C. H. Yoon, C. Kupitz, R. G. Sierra, E. H. Snell, H. De Mirci, Near-physiological-temperature serial crystallography reveals conformations of sars-cov-2 main protease active site for improved drug repurposing. *Structure* **29**, 1382–1396.e6 (2021).
34. T. T. Tanimoto, Elementary mathematical theory of classification and prediction (1958).
35. D. Rogers, M. Hahn, Extended-connectivity fingerprints. *J. Chem. Inf. Model.* **50**, 742–754 (2010).
36. A. Daina, O. Michielin, V. Zoete, Swissadme: A free web tool to evaluate pharmacokinetics, drug-likeness and medicinal chemistry friendliness of small molecules. *Sci. Rep.* **7**, 1–13 (2017).
37. S. C. Hoffman, V. Chenthamarakshan, K. Wadhawan, P.-Y. Chen, P. Das, Optimizing molecules using efficient queries from property evaluations. *Nat. Mach. Intell.* **4**, 21–31 (2022).
38. A. Luttens, H. Gullberg, E. Abdurakhmanov, D. D. Vo, D. Akaberi, V. O. Talibov, N. Nekhotiaeva, L. Vangeel, S. De Jonghe, D. Jochmans, J. Krambrich, A. Tas, B. Lundgren, Y. Gravenfors, A. J. Craig, Y.

Atilaw, A. Sandström, L. W. K. Moodie, Å. Lundkvist, M. J. van Hemert, J. Neyts, J. Lennerstrand, J. Kihlberg, K. Sandberg, U. H. Danielson, J. Carlsson, Ultralarge virtual screening identifies sars-cov-2 main protease inhibitors with broad-spectrum activity against coronaviruses. *J. Am. Chem. Soc.* **144**, 2905–2920 (2022).

39. The COVID Moonshot Consortium, H. Achdout, A. Aimon, E. Bar-David, H. Barr, A. Ben-Shmuel, J. Bennett, M. L. Bobby, J. Brun, BVNBS Sarma, M. Calmiano, A. Carbery, E. Cattermole, J. D. Chodera, A. Clyde, J. E. Coffland, G. Cohen, J. Cole, A. Contini, L. Cox, M. Cvitkovic, A. Dias, A. Douangamath, S. Duberstein, T. Dudgeon, L. Dunnett, P. K. Eastman, N. Erez, M. Fairhead, D. Fearon, O. Fedorov, M. Ferla, H. Foster, R. Foster, R. Gabizon, P. Gehrtz, C. Gileadi, C. Giroud, W. G. Glass, R. Glen, I. Glinert, M. Gorichko, T. Gorrie-Stone, E. J. Griffen, J. Heer, M. Hill, S. Horrell, M. F. D. Hurley, T. Israely, A. Jajack, E. Jnoff, T. John, A. L. Kantsadi, P. W. Kenny, J. L. Kiappes, L. Koekemoer, B. Kovar, T. Krojer, A. A. Lee, B. A. Lefker, H. Levy, N. London, P. Lukacik, H. B. Macdonald, B. M. Lean, T. R. Malla, T. Matviiuk, W. M. Corkindale, S. Melamed, O. Michurin, H. Mikolajek, A. Morris, G. M. Morris, M. J. Morwitzer, D. Moustakas, J. B. Neto, V. Oleinikovas, G. J. Overheul, D. Owen, R. Pai, J. Pan, N. Paran, B. Perry, M. Pingle, J. Pinjari, B. Politi, A. Powell, V. Psenak, R. Puni, V. L. Rangel, R. N. Reddi, S. P. Reid, E. Resnick, M. C. Robinson, R. P. Robinson, D. Rufa, C. Schofield, A. Shaikh, J. Shi, K. Shurrush, A. Sittner, R. Skyner, A. Smalley, M. D. Smilova, J. Spencer, C. Strain-Damerell, V. Swamy, H. Tamir, R. Tennant, A. Thompson, W. Thompson, S. Tomasio, A. Tumber, I. Vakonakis, R. P. van Rij, F. S. Varghese, M. Vaschetto, E. B. Vitner, V. Voelz, Annette von Delft, Frank von Delft, M. Walsh, W. Ward, C. Weatherall, S. Weiss, C. F. Wild, M. Wittmann, N. Wright, Y. Yahalom-Ronen, D. Zaidmann, H. Zidane, N. Zitzmann, Open science discovery of potent non-covalent SARS-CoV-2 main protease inhibitors. bioRxiv 2020.10.29.339317 [**Preprint**] (2 March 2020).

40. Y. Unoh, S. Uehara, K. Nakahara, H. Nobori, Y. Yamatsu, S. Yamamoto, Y. Maruyama, Y. Taoda, K. Kasamatsu, T. Suto, K. Kouki, A. Nakahashi, S. Kawashima, T. Sanaki, S. Toba, K. Uemura, T. Mizutare, S. Ando, M. Sasaki, Y. Orba, H. Sawa, A. Sato, T. Sato, T. Kato, Y. Tachibana, Discovery of s-217622, a non-covalent oral sars-cov-2 3cl protease inhibitor clinical candidate for treating covid-19. bioRxiv 2022.01.26.477782 [**Preprint**] (26 January 2022).

41. F. Ren, X. Ding, M. Zheng, M. Korzinkin, X. Cai, W. Zhu, A. Mantsyzov, A. Aliper, V. Aladinskiy, Z. Cao, S. Kong, X. Long, B. H. M. Liu, Y. Liu, V. Naumov, A. Shneyderman, I. V. Ozerov, J. Wang, F. W. Pun, A. Aspuru-Guzik, M. Levitt, A. Zhavoronkov, Alphafold accelerates artificial intelligence

powered drug discovery: Efficient discovery of a novel cyclindependent kinase 20 (cdk20) small molecule inhibitor. arXiv:2201.09647 [q-bio.BM] (21 January 2022).

42. M. Baek, F. D. Maio, I. Anishchenko, J. Dauparas, S. Ovchinnikov, G. R. Lee, J. Wang, Q. Cong, L. N. Kinch, R. D. Schaeffer, C. Millán, H. Park, C. Adams, C. R. Glassman, A. De Giovanni, J. H. Pereira, A. V. Rodrigues, A. A. van Dijk, A. C. Ebrecht, D. J. Opperman, T. Sagmeister, C. Buhlheller, T. Pavkov-Keller, M. K. Rathinaswamy, U. Dalwadi, C. K. Yip, J. E. Burke, K. C. Garcia, N. V. Grishin, P. D. Adams, R. J. Read, D. Baker, Accurate prediction of protein structures and interactions using a three-track neural network. *Science* **373**, 871–876 (2021).
43. D. Merk, F. Grisoni, L. Friedrich, L. G. Schneider, Tuning artificial intelligence on the de novo design of natural-productinspired retinoid x receptor modulators. *Commun. Chem.* **1**, 1–9 (2018).
44. D. Polykovskiy, A. Zhebrak, D. Vetrov, Y. Ivanenkov, V. Aladinskiy, P. Mamoshina, M. Bozdaganyan, A. Aliper, A. Zhavoronkov, A. Kadurin, Entangled conditional adversarial autoencoder for de novo drug discovery. *Mol. Pharm.* **15**, 4398–4405 (2018).
45. E. Putin, A. Asadulaev, Q. Vanhaelen, Y. Ivanenkov, A. V. Aladinskaya, A. Aliper, A. Zhavoronkov, Adversarial threshold neural computer for molecular de novo design. *Mol. Pharm.* **15**, 4386–4397 (2018).
46. X. Tan, X. Jiang, Y. He, F. Zhong, X. Li, Z. Xiong, Z. Li, X. Liu, C. Cui, Q. Zhao, Y. Xie, F. Yang, C. Wu, J. Shen, M. Zheng, Z. Wang, H. Jiang, Automated design and optimization of multitarget schizophrenia drug candidates by deep learning. *Eur. J. Med. Chem.* **204**, 112572 (2020).
47. M. Assmann, M. Bal, M. Craig, J. D'Oyley, L. Phillips, H. Triendl, P. A. Bates, U. Bashir, P. Ruprah, N. Shaker, V. Stojevic, A novel machine learning approach uncovers new and distinctive inhibitors for cyclin-dependent kinase 9. bioRxiv 2020.03.18.996538 [**Preprint**] (19 March 2020).
48. A. Rives, J. Meier, T. Sercu, S. Goyal, Z. Lin, J. Liu, D. Guo, M. Ott, C Lawrence Zitnick, J. Ma, R. Fergus, Biological structure and function emerge from scaling unsupervised learning to 250 million protein sequences. *Proc. Natl. Acad. Sci. U.S.A.* **118**, e2016239118 (2021).
49. W. Dejnirattisai, J. Huo, D. Zhou, J. Zahradník, P. Supasa, C. Liu, H. M. E. Duyvesteyn, H. M. Ginn, A. J. Mentzer, A. Tuekprakhon, R. Nutalai, B. Wang, A. Dijokaite, S. Khan, O. Avinoam, M. Bahar, D. Skelly, S. Adele, S. A. Johnson, A. Amini, T. G. Ritter, C. Mason, C. Dold, D. Pan, S. Assadi, A. Bellass, N. Omo-Dare, D. Koeckerling, A. Flaxman, D. Jenkin, P. K. Aley, M. Voysey, S. A. C. Clemens, F. G. Naveca, V. Nascimento, F. Nascimento, C. F. da Costa, P. C. Resende, A. Pauvolid-Correa, M. M. Siqueira, V. Baillie, N. Serafin, G. Kwatra, K. D. Silva, S. A. Madhi, M. C. Nunes, T.

Malik, P. J. M. Openshaw, J. K. Baillie, M. G. Semple, A. R. Townsend, K.-Y. A. Huang, T. K. Tan, M. W. Carroll, P. Klenerman, E. Barnes, S. J. Dunachie, B. Constantinides, H. Webster, D. Crook, A. J. Pollard, T. Lambe; OPTIC Consortium; ISARICC Consortium; N. G. Paterson, M. A. Williams, D. R. Hall, E. E. Fry, J. Mongkolsapaya, J. Ren, G. Schreiber, D. I. Stuart, G. R. Screaton, Sars-cov-2 omicron-b. 1.1. 529 leads to widespread escape from neutralizing antibody responses. *Cell* **185**, 467–484.e15 (2022).

50. S. C. Hoffman, V. Chenthamarakshan, D. Zubarev, D. P. Sanders, P. Das, Sample-efficient generation of novel photo-acid generator molecules using a deep generative model, in *NeurIPS 2021 Workshop on Deep Generative Models and Downstream Applications* (2021).
51. N. W. Gebauer, M. Gastegger, S. S. Hessmann, K.-R. Muller, K. T. Schutt, Inverse design of 3d molecular structures with conditional generative neural networks. *Nat. Commun.* **13**, 1–11 (2022).
52. Y. Schiff, V. Chenthamarakshan, S. Hoffman, K. N. Ramamurthy, P. Das, Augmenting molecular deep generative models with topological data analysis representations, in *IEEE International Conference on Acoustics, Speech, and Signal Processing* (IEEE, 2022).
53. J. Ross, B. Belgodere, V. Chenthamarakshan, I. Padhi, Y. Mroueh, P. das, Large-scale chemical language representations capture molecular structure and properties. *Nat. Mach. Intell.* **4**, 1256–1264 (2022).
54. S. R. Bowman, L. Vilnis, O. Vinyals, A. M. Dai, R. Jozefowicz, S. Bengio, Generating sentences from a continuous space. arXiv:1511.06349 [cs.LG] (19 Novenber 2015).
55. D. Polykovskiy, A. Zhebrak, B. Sanchez-Lengeling, S. Golovanov, O. Tatanov, S. Belyaev, R. Kurbanov, A. Artamonov, V. Aladinskiy, M. Veselov, A. Kadurin, S. Johansson, H. Chen, S. Nikolenko, A. Aspuru-Guzik, A. Zhavoronkov, Molecular sets (MOSES): A benchmarking platform for molecular generation models. arXiv:1811.12823 [cs.LG] (29 November 2018).
56. J. J. Irwin, B. K. Shoichet, ZINC–A free database of commercially available compounds for virtual screening. *J. Chem. Inf. Model.* **45**, 177–182 (2005).
57. R. Gómez-Bombarelli, J. N. Wei, D. Duvenaud, J. M. Hernández-Lobato, B. Sánchez-Lengeling, D. Sheberla, J. Aguilera-Iparraguirre, T. D. Hirzel, R. P. Adams, A. Aspuru-Guzik, Automatic chemical design using a data-driven continuous representation of molecules. *ACS Cent. Sci.* **4**, 268–276 (2018).
58. M. K. Gilson, T. Liu, M. Baitaluk, G. Nicola, L. Hwang, J. Chong, BindingDB in 2015: A public database for medicinal chemistry, computational chemistry and systems pharmacology. *Nucleic Acids Res.* **44**, D1045–D1053 (2015).

59. M. Karimi, D. Wu, Z. Wang, Y. Shen, Deepaffinity: Interpretable deep learning of compound–Protein affinity through unified recurrent and convolutional neural networks. *Bioinformatics* **35**, 3329–3338 (2019).
60. RDKit: Open-source cheminformatics; http://www.rdkit.org [accessed 7 March 2022].
61. K. W. Lim, B. Sharma, P. Das, V. Chenthamarakshan, J. S. Dordick, Explaining chemical toxicity using missing features. arXiv:2009.12199 [q-bio.QM] (23 September 2020).
62. R. Huang, M. Xia, D.-T. Nguyen, T. Zhao, S. Sakamuru, J. Zhao, S. A. Shahane, A. Rossoshek, A. Simeonov, Tox21 challenge to build predictive models of nuclear receptor and stress response pathways as mediated by exposure to environmental chemicals and drugs. *Front. Environ. Sci.* **3**, 85 (2016).
63. Z. Wu, B. Ramsundar, E. N. Feinberg, J. Gomes, C. Geniesse, A. S. Pappu, K. Leswing, V. Pande, Moleculenet: A benchmark for molecular machine learning. *Chem. Sci.* **9**, 513–530 (2018).
64. O. Trott, A. J. Olson, AutoDock Vina: Improving the speed and accuracy of docking with a new scoring function, efficient optimization, and multithreading. *J. Comput. Chem.* **31**, 455–461 (2010).
65. L. Schrodinger, The PyMOL molecular graphics system, version 2.4.1 (2020).
66. E. F. Pettersen, T. D. Goddard, C. C. Huang, E. C. Meng, G. S. Couch, T. I. Croll, J. H. Morris, T. E. Ferrin, Ucsf chimerax: Structure visualization for researchers, educators, and developers. *Protein Sci.* **30**, 70–82 (2021).
67. R. A. Laskowski, M. B. Swindells, Ligplot+: Multiple ligand-protein interaction diagrams for drug discovery. *J. Chem. Inf. Model.* **51**, 2778–2786 (2011).
68. S. Chatterjee, P. G. Debenedetti, F. H. Stillinger, R. M. Lynden-Bell, A computational investigation of thermodynamics, structure, dynamics and solvation behavior in modified water models. *J. Chem. Phys.* **128**, 124511 (2008).
69. M. J. Robertson, J. Tirado-Rives, W. L. Jorgensen, Improved peptide and protein torsional energetics with the OPLS-AA force field. *J. Chem. Theory Comput.* **11**, 3499–3509 (2015).
70. P. Bauer, B. Hess, E. Lindahl, Gromacs 2022.4 manual (2022); 10.5281/zenodo.7323409.
71. eMolecules, eMolecules plus database (2020); https://www.emolecules.com/. [accessed May 2020].
72. X. Xue, H. Yang, W. Shen, Q. Zhao, J. Li, K. Yang, C. Chen, Y. Jin, M. Bartlam, Z. Rao, Production of authentic sars-cov mpro with enhanced activity: Application as a novel tag-cleavage endopeptidase for protein overproduction. *J. Mol. Biol.* **366**, 965–975 (2007).
73. A. Douangamath, D. Fearon, P. Gehrtz, T. Krojer, P. Lukacik, C. David Owen, E. Resnick, C. Strain-Damerell, A. Aimon, P. Ábrányi-Balogh, J. Brandão-Neto, A. Carbery, G. Davison, A. Dias, T. D.

Downes, L. Dunnett, M. Fairhead, J. D. Firth, S. Paul Jones, A. Keeley, G. M. Keserü, H. F. Klein, M. P. Martin, M. E. M. Noble, M. A. Walsh, Crystallographic and electrophilic fragment screening of the SARS-CoV-2 main protease. *Nat. Commun.* **11**, 5047 (2020).

74. D. Wrapp, N. Wang, K. S. Corbett, J. A. Goldsmith, C.-L. Hsieh, O. Abiona, B. S. Graham, Jason S. McLellan, Cryo-em structure of the 2019-ncov spike in the prefusion conformation. *Science* **367**, 1260–1263 (2020).
75. A. Douangamath, A. Powell, D. Fearon, P. M. Collins, R. Talon, T. Krojer, R. Skyner, J. Brandao-Neto, L. Dunnett, A. Dias, A. Aimon, N. M. Pearce, C. Wild, T. Gorrie-Stone, F. von Delft, Achieving efficient fragment screening at XChem facility at diamond light source. *J. Vis. Exp.* e62414 (2021).
76. T. Krojer, R. Talon, N. Pearce, P. Collins, A. Douangamath, J. Brandao-Neto, A. Dias, B. Marsden, F. von Delft, The xchemexplorer graphical workflow tool for routine or large-scale protein–ligand structure determination. *Acta Crystallogr. Sect. D Struct. Biol.* **73**, 267–278 (2017).
77. M. D. Winn, C. C. Ballard, K. D. Cowtan, E. J. Dodson, P. Emsley, P. R. Evans, R. M. Keegan, E. B. Krissinel, A. G. W. Leslie, A. McCoy, S. J. McNicholas, G. N. Murshudov, N. S. Pannu, E. A. Potterton, H. R. Powell, R. J. Read, A. Vagin, K. S. Wilson, Overview of the ccp4 suite and current developments. *Acta Crystallogr. Sect. D Biol. Crystallogr.* **67**, 235–242.
78. N. M. Pearce, T. Krojer, A. R. Bradley, P. Collins, R. P. Nowak, R. Talon, B. D. Marsden, S. Kelm, J. Shi, C. M. Deane, F. von Delft, A multi-crystal method for extracting obscured crystallographic states from conventionally uninterpretable electron density. *Nat. Commun.* **8**, 15123 (2017).
79. P. Emsley, B. Lohkamp, W. G. Scott, K. Cowtan, Features and development of coot. *Acta Crystallogr. Sect. D Biol. Crystallogr.* **66**, 486–501 (2010).
80. F. Long, R. A. Nicholls, P. Emsley, S. Gražulis, A. Merkys, A. Vaitkus, G. N. Murshudov, AceDRG: A stereochemical description generator for ligands. *Acta Crystallogr. Sect. D Struct. Biol.* **73**, 112–122 (2017).
81. O. S. Smart, *et al.* grade, version 1.2.20 (2021); https://www.globalphasing.com/.
82. G. N. Murshudov, A. A. Vagin, E. J. Dodson, Refinement of macromolecular structures by the maximum-likelihood method. *Acta Crystallogr. Sect. D Biol. Crystallogr.* **53**, 240–255 (1997).
83. G. Bricogne, *et al.* Buster, version 2.10.4 (Global Phasing Ltd., 2017).
84. T. Zhou, Y. Tsybovsky, J. Gorman, M. Rapp, G. Cerutti, G.Y. Chuang, P. S. Katsamba, J. M. Sampson, A. Schön, J. Bimela, J. C. Boyington, A. Nazzari, A. S. Olia, W. Shi, M. Sastry, T. Stephens, J. Stuckey, I.T. Teng, P. Wang, S. Wang, B. Zhang, R. A. Friesner, D. D. Ho, J. R. Mascola, L. Shapiro, P. D.

Kwong, Cryo-EM structures of SARS-CoV-2 spike without and with ACE2 reveal a pH-dependent switch to mediate endosomal positioning of receptor-binding domains. *Cell Host Microbe* **28**, 867–879.e5 (2020).

85. P. Bond, JavaScript Thermal Shift Analysis Software; https://paulsbond.co.uk/jtsa. [accessed 7 March 2022].

86. The COVID Moonshot Consortium, H. Achdout, A. Aimon, E. Bar-David, H. Barr, A. Ben-Shmuel, J. Bennett, M. L. Boby, B. Borden, G. R. Bowman, J. Brun, S. Bvnbs, M. Calmiano, A. Carbery, E. Cattermole, E. Chernyshenko, J. D. Chodera, A. Clyde, J. E. Coffland, G. Cohen, J. Cole, A. Contini, L. Cox, M. Cvitkovic, A. Dias, K. Donckers, D. L. Dotson, A. Douangamath, S. Duberstein, T. Dudgeon, L. Dunnett, P. K. Eastman, N. Erez, C. J. Eyermann, M. Fairhead, G. Fate, D. Fearon, O. Fedorov, M. Ferla, R. S. Fernandes, L. Ferrins, R. Foster, H. Foster, R. Gabizon, A. Garcia-Sastre, V. O. Gawriljuk, P. Gehrtz, C. Gileadi, C. Giroud, W. G. Glass, R. Glen, I. Glinert, A. S. Godoy, M. Gorichko, T. Gorrie-Stone, E. J. Griffen, S. H. Hart, J. Heer, M. Henry, M. Hill, S. Horrell, M. F. Hurley, T. Israely, A. Jajack, E. Jnoff, D. Jochmans, T. John, S. D. Jonghe, A. L. Kantsadi, P. W. Kenny, J. L. Kiappes, L. Koekemoer, B. Kovar, T. Krojer, A. A. Lee, B. A. Lefker, H. Levy, N. London, P. Lukacik, H. B. Macdonald, B. MacLean, T. R. Malla, T. Matviiuk, W. McCorkindale, B. L. McGovern, S. Melamed, O. Michurin, H. Mikolajek, B. F. Milne, A. Morris, G. M. Morris, M. J. Morwitzer, D. Moustakas, A. M. Nakamura, J. B. Neto, J. Neyts, L. Nguyen, G. D. Noske, V. Oleinikovas, G. Oliva, G. J. Overheul, D. Owen, V. Psenak, R. Pai, J. Pan, N. Paran, B. Perry, M. Pingle, J. Pinjari, B. Politi, A. Powell, R. Puni, V. L. Rangel, R. N. Reddi, S. P. Reid, E. Resnick, E. G. Ripka, M. C. Robinson, R. P. Robinson, J. Rodriguez-Guerra, R. Rosales, D. Rufa, C. Schofield, M. Shafeev, A. Shaikh, J. Shi, K. Shurrush, S. Singh, A. Sittner, R. Skyner, A. Smalley, M. D. Smilova, L. J. Solmesky, J. Spencer, C. Strain-Damerell, V. Swamy, H. Tamir, R. Tennant, W. Thompson, A. Thompson, W. Thompson, S. Tomasio, A. Tumber, I. Vakonakis, R. P. van Rij, L. Vangeel, F. S. Varghese, M. Vaschetto, E. B. Vitner, V. Voelz, A. Volkamer, F. von Delft, A. von Delft, M. Walsh, W. Ward, C. Weatherall, S. Weiss, K. M. White, C. F. Wild, M. Wittmann, N. Wright, Y. Yahalom-Ronen, D. Zaidmann, H. Zidane, and N. Zitzmann, Open science discovery of oral non-covalent SARS-CoV-2 main protease inhibitor therapeutics. bioRxiv 2020.10.29.339317 [**Preprint**] (30 January 2022).

87. M. Sasaki, K. Tabata, M. Kishimoto, Y. Itakura, H. Kobayashi, T. Ariizumi, K. Uemura, S. Toba, S. Kusakabe, Y. Maruyama, S. Iida, N. Nakajima, T. Suzuki, S. Yoshida, H. Nobori, T. Sanaki, T. Kato, T. Shishido, W. W. Hall, Y. Orba, A. Sato, H. Sawa, Oral administration of S-217622, a SARS-CoV-2

main protease inhibitor, decreases viral load and accelerates recovery from clinical aspects of COVID-19. bioRxiv 2022.02.14.480338 [**Preprint**] (15 February 2022).

88. W. Fischer, J. J. Eron, W. Holman, M. S. Cohen, L. Fang, L. J. Szewczyk, T. P. Sheahan, R. Baric, K. R. Mollan, C. R. Wolfe, E. R. Duke, M. M. Azizad, K. Borroto-Esoda, D. A. Wohl, A. J. Loftis, P. Alabanza, F. Lipansky, W. P. Painter, Molnupiravir, an oral antiviral treatment for COVID-19. medRxiv 2021.06.17.21258639 [**Preprint**] (17 June 2021).
89. C. A. Lipinski, Lead-and drug-like compounds: The rule-of-five revolution. *Drug Discov. Today Technol.* **1**, 337–341 (2004).
90. A. K. Ghose, V. N. Viswanadhan, J. J. Wendoloski, A knowledge-based approach in designing combinatorial or medicinal chemistry libraries for drug discovery. 1. A qualitative and quantitative characterization of known drug databases. *J. Comb. Chem.* **1**, 55–68 (1999).
91. D. F. Veber, S. R. Johnson, H.-Y. Cheng, B. R. Smith, K. W. Ward, K. D. Kopple, Molecular properties that influence the oral bioavailability of drug candidates. *J. Med. Chem.* **45**, 2615–2623 (2002).
92. W. J. Egan, K. M. Merz, J. J. Baldwin, Prediction of drug absorption using multivariate statistics. *J. Med. Chem.* **43**, 3867–3877 (2000).
93. I. Muegge, S. L. Heald, D. Brittelli, Simple selection criteria for drug-like chemical matter. *J. Med. Chem.* **44**, 1841–1846 (2001).
94. Y. C. Martin, A bioavailability score. *J. Med. Chem.* **48**, 3164–3170 (2005).
95. J. B. Baell, G. A. Holloway, New substructure filters for removal of pan assay interference compounds (pains) from screening libraries and for their exclusion in bioassays. *J. Med. Chem.* **53**, 2719–2740 (2010).
96. R. Brenk, A. Schipani, D. James, A. Krasowski, I. H. Gilbert, J. Frearson, P. G. Wyatt, Lessons learnt from assembling screening libraries for drug discovery for neglected diseases. *ChemMedChem* **3**, 435–444 (2008).
97. S. J. Teague, A. M. Davis, P. D. Leeson, T. Oprea, The design of leadlike combinatorial libraries. *Angew. Chem. Int. Ed.* **38**, 3743–3748 (1999).
98. M. H. Segler, T. Kogej, C. Tyrchan, M. P. Waller, Generating focused molecule libraries for drug discovery with recurrent neural networks. *ACS Cent. Sci.* **4**, 120–131 (2017).

99. A. Kadurin, A. Aliper, A. Kazennov, P. Mamoshina, Q. Vanhaelen, K. Khrabrov, A. Zhavoronkov, The cornucopia of meaningful leads: Applying deep adversarial autoencoders for new molecule development in oncology. *Oncotarget* **8**, 10883–10890 (2017).

100. W. Jin, R. Barzilay, T. Jaakkola, Junction tree variational autoencoder for molecular graph generation. arXiv:1802.04364 (2018).

101. O. Prykhodko, S. V. Johansson, P.-C. Kotsias, J. Arús-Pous, E. J. Bjerrum, O. Engkvist, H. Chen, A de novo molecular generation method using latent vector based generative adversarial network. *J. Cheminform.* **11**, 74 (2019).
